# Tumor cell plasticity in targeted therapy-induced resistance: mechanisms and new strategies

**DOI:** 10.1038/s41392-023-01383-x

**Published:** 2023-03-11

**Authors:** Zhen-Duo Shi, Kun Pang, Zhuo-Xun Wu, Yang Dong, Lin Hao, Jia-Xin Qin, Wei Wang, Zhe-Sheng Chen, Cong-Hui Han

**Affiliations:** 1 Department of Urology, Xuzhou Clinical School of Xuzhou Medical University, Jiangsu, China; 2 Department of Urology, Xuzhou Central Hospital, Xuzhou, Jiangsu China; 3 School of Life Sciences, Jiangsu Normal University, Jiangsu, China; 4 Department of Urology, Heilongjiang Provincial Hospital, Heilongjiang, China; 5 Department of Pharmaceutical Sciences, College of Pharmacy and Health Sciences, St. John’s University, Queens, NY 11439 USA; 6 Department of Medical College, Southeast University, Nanjing, China

**Keywords:** Cancer therapy, Tumour heterogeneity

## Abstract

Despite the success of targeted therapies in cancer treatment, therapy-induced resistance remains a major obstacle to a complete cure. Tumor cells evade treatments and relapse via phenotypic switching driven by intrinsic or induced cell plasticity. Several reversible mechanisms have been proposed to circumvent tumor cell plasticity, including epigenetic modifications, regulation of transcription factors, activation or suppression of key signaling pathways, as well as modification of the tumor environment. Epithelial-to-mesenchymal transition, tumor cell and cancer stem cell formation also serve as roads towards tumor cell plasticity. Corresponding treatment strategies have recently been developed that either target plasticity-related mechanisms or employ combination treatments. In this review, we delineate the formation of tumor cell plasticity and its manipulation of tumor evasion from targeted therapy. We discuss the non-genetic mechanisms of targeted drug-induced tumor cell plasticity in various types of tumors and provide insights into the contribution of tumor cell plasticity to acquired drug resistance. New therapeutic strategies such as inhibition or reversal of tumor cell plasticity are also presented. We also discuss the multitude of clinical trials that are ongoing worldwide with the intention of improving clinical outcomes. These advances provide a direction for developing novel therapeutic strategies and combination therapy regimens that target tumor cell plasticity.

## Introduction to tumor cell plasticity (TCP)

Cellular plasticity is a fundamental feature of cells. During development, cells progressively differentiate into different hierarchies and obtain specific developmental commitments. Such processes are reversible, and the phenotypic changes can be diversely directed as needed. Genetic lineage tracing has demonstrated that functionally mature cells can undergo dedifferentiation (lineage reversion), transdifferentiation (cells converting into other distant lineages), or transdetermination (cells switching lineage commitment to a closely related cell type) upon injury or disease.^1,2^ As far back as 1956, Conrad Waddington stated that during development embryonic progenitor cells progressively differentiate into distinct cell types and are increasingly restricted within a specific lineage.^3^ The final specialized cells are then integrated into their respective tissues and conduct their specific invariable functions. However, later studies subverted this paradigm. Han Driesch isolated from a sea urchin embryo a single blastomere in an early multicellular state could develop into an entire larva.^4^ This finding suggested that partially developed cells are not fully determined but retain the potency to produce all types of cells found in an entire organism.

In fact, the states of cells are quite dynamic, especially when injury or disease occurs. An equilibrium between new cell generation and old cell loss is generally maintained in multicellular organisms. If perturbed by unexpected loss, stem cell replication and differentiation along with transformation of mature differentiated cells can restore equilibrium. For example, endothelial cells assume arterial, venous, or lymphatic identity in an early developmental stage. However, upon injury, endothelial cells can undergo lineage changes to become hematopoietic progenitor or cardiac valve cells.^5–7^ In liver homeostasis, the modest proliferation of hepatocytes in all zones contributes to the homeostatic hepatocyte pool, rather than a rare type of liver progenitor cell (LPC) subpopulation.^8,9^ In a genetic mouse model which mimics human Alagille syndrome and contributes to biliary epithelium regeneration, hepatocytes are able to transdifferentiate into biliary epithelial cells.^10^ Additionally, cellular plasticity can be “artificially” enhanced, as demonstrated by induced pluripotent stem cells (iPSCs), which were first generated in 2007,^11^ providing unprecedented opportunities for novel therapies.

**Fig. 1:**
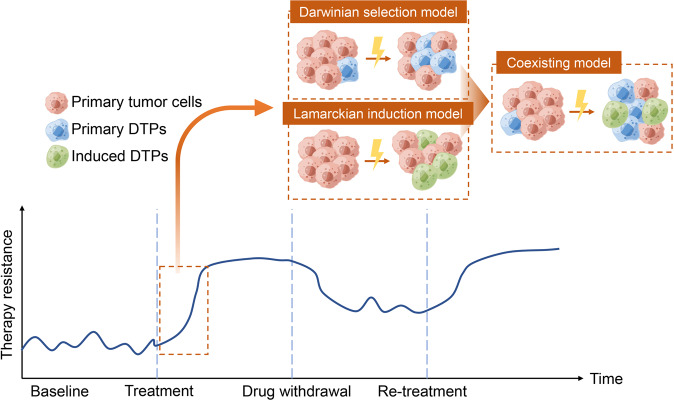
Changes in acquired therapy resistance induced by treatment initiation and discontinuation, with different scenarios employed to interpret the development of the therapy-resistant phenotype. According to the Darwinian selection model, drug-tolerant persistors (DTPs) that originated from the primary tumor tissue are selected and enriched by treatments. The Lamarckian induction model regards the acquired drug-indifferent phenotype as the result of tumor cell adaptation to treatments that leads to formation of induced DTPs. The coexisting model suggests that formation of both primary and induced DTPs occur and together contribute to the acquired therapy resistance

Cellular plasticity also contributes to pathological conditions. Similar to the cell plasticity observed during post-injury repair and regeneration, plasticity in tumor cells promotes survival against the immune attack and anti-tumor treatments. A major obstacle preventing complete remission, this evasion to therapy occurs as cancer cells play “hide and seek” by constant phenotypic switching. Some regard this phenotypic switching of cancer cells to result from passive Darwinian selection and enrichment upon drug exposure,^12,13^ while others argue it is an active adaptation to internal or external stimuli (Lamarckian induction).^14^ A coexisting model has also been formulated to illustrate that these two concepts complement and intertwine with each other (Fig. 1).^14–16^ The “hide and seek” behavior of cancer cells is known as TCP, which is a process that re-activates various developmental programs to achieve phenotypic switching. TCP enables transformation of tumor cell identity that allows the cells to adapt to different microenvironments and circumvent the drug-targeted pathway. This subpopulation of tumor cells is characterized by its dormant and stem-like trait and termed drug-tolerant persisters (DTPs).^15^ The “acquired inertia” of DTPs is regarded as a survival skill exposed by antitumor treatment that can fade away after a drug-free interval.^17^ Thus, with TCP, a reversible switching of phenotypes can be achieved between proliferative, metastatic tumor cells and dormant, drug-indifferent DTPs, thereby hindering the desired outcome of targeted therapies. By this universally plastic behavior, tumor cells transform in different but functionally overlapping processes, including epithelial-mesenchymal transition (EMT), transdifferentiation, and acquiring properties of cancer stem cells (CSCs).^14,18,19^

### Epithelial–mesenchymal transition

EMT is a process by which tumor cells lose their epithelial features and acquire mesenchymal characteristics. EMT is characterized by loss of apical-basal polarity and disruption of intercellular interactions, accompanied by the acquisition of front-rear polarity and remodeling of the cytoskeleton.^20,21^ On the other hand, mesenchymal-epithelial transition (MET) is the inverse process of EMT. EMT and MET are thought to be regulated mainly at the transcriptional level by the activity of EMT transcription factors (EMT-TFs), including Snail, Slug, Zeb1/2, Twist, and microRNAs (miRs). Signaling pathways such as transforming growth factor (TGF-β), wingless/integrated (WNT), NOTCH, and HIPPO are also involved.^15,22^ Two double-negative feedback loops that include Snail/miR-34 and Zeb/miR-200 help to manipulate the initiation, transition, and maintenance of EMT program.^23,24^ Genetic mutations may also play a role in EMT regulation. As indicated by Martin et al.,^25^ a combined loss of the tumor suppressor gene *P53* and *PTEN* in clonal prostate epithelial cells can lead to transformation of multipotent progenitors and ultimately EMT. Yamasaki et al.^26^ recently proposed that Kirsten rat sarcoma viral oncogene homolog (*KRAS*) mutation can also promote an EMT-like phenotypic change.

Both EMT and MET have been proposed to play key roles in cancer progression.^21^ Apart from a complete EMT/MET transition, tumor cells can exhibit a “partial EMT” state, a hybrid of epithelial and mesenchymal phenotypes.^27^ In fact, tumor cells can exhibit a spectrum of EMT states characterized by varying proportions of epithelial and mesenchymal traits, both in circulation and at a secondary site.^28,29^ Panchy et al.^30^ recently confirmed this epithelial-mesenchymal spectrum using transcriptomic analysis. This hybrid epithelial-mesenchymal state enables tumor cells to self-adapt and adjust quickly to changing environments, thus representing an ideal mechanism of TCP.^28^ Previous studies indicate that dynamic changes between epithelial and mesenchymal phenotype in tumor cells with partial EMT are critically important to the metastatic potential and therapy resistance of tumor cells.^31^

The contributions made by EMT programs to metastasis were first proposed by Yang et al.,^32^ who described that inhibition of Twist, a classic EMT-TF, can significantly restrain highly metastatic mammary carcinoma cells from metastasizing to the lung. The impact of other EMT-TFs on metastasis was then extensively investigated in different types of cancers.^33^ For example, depletion of Zeb1 was found to markedly inhibit pancreatic ductal adenocarcinoma progression towards metastatic type.^34^ Zeb1 was also found to function as a key master regulator of multiple EMT-TFs and metastasis in breast cancer.^33^ The MET program, in contrast, presumably functions during metastasis when the migratory CSCs settle down and adapt to a new microenvironment. In the process of colonization, the migratory CSCs regain self-renewal capacity and spawn fast-cycling epithelial progeny, thereby restoring epithelial characteristics.^35^ Such reversible EMT-MET programs seem to be a common feature shared by different cancer types during the seeding of a secondary tumor.^35–37^

Evidence also suggests that EMT can serve as a survival mechanism for cancer cells to escape immunosurveillance and resist therapy-induced death.^38^ Multiple studies have observed a strong correlation between the level of mesenchymal cell markers and therapeutic resistance.^21,39–42^ For example, Farmer et al.^42^ demonstrated that an upregulation of stromal gene expression predicted resistance to preoperative chemotherapy with 5-fluororacil, epirubicin, and cyclophosphamide in estrogen receptor (ER)-negative breast cancer. In ovarian cancer, inhibition of EMT can effectively eliminate cisplatin resistance.^43^ Byers et al.^40^ revealed a correlation between a 76-gene mesenchymal signature and non-small-cell lung cancer (NSCLC) resistance to epidermal growth factor receptor-tyrosine kinase inhibitors (EGFR-TKIs) and phosphatidylinositol 3-kinase/protein kinase B (PI3K/Akt) inhibitors. By proteomic screening, Paulitschke et al.^41^ also found that two EMT-related markers are effective in determining drug susceptibility in melanoma cells. Although the mechanisms underlying the therapeutic resistance conferred by EMT remain unclear, several hypotheses have been put forward, including reduced levels of proapoptotic proteins, increased drug efflux, and immunosuppressive tumor microenvironment (TME).^44–46^

### Transdifferentiation

Transdifferentiation, also known as lineage plasticity or lineage switching, is a radical shift in cell identity that leads to cells acquiring new transcriptional or morphological characteristics.^47,48^ In the context of cancer, such lineage plasticity may serve as a promising therapeutic approach. For example, melanoma spheroid cells are able to transdifferentiate into benign cell lineages including melanocytes, adipocytes, osteocytes and chondrocytes.^49^ However, transdifferentiation in cancer cells can also be a double-edge sword. Transdifferentiation observed in basal cell carcinoma (BCC) contributes to BCC relapse following treatment discontinuation.^50,51^ When treated with vismodegib, some BCC cells transitioned from a phenotype featuring a transcriptional bulge-like signature to a mixed isthmus/interfollicular epidermis cell phenotype, thereby achieving drug resistance.^52^ A discontinuation of treatment led to tumor cell reactivation and resumption of proliferation.^52,53^ Another distinctive form of transdifferentiation is characterized by a drug-induced neuroendocrine transdifferentiation (NET). Observed in prostate cancer (PC) and lung adenocarcinoma (LUAD), NET induces drug resistance, thereby causing treatment failure and relapse.^14,54,55^ Here, we consider PC and LUAD as two typical examples of NET for further discussion.

Through NET, PC tumor cells gradually obtain the histomorphology of small cell prostate cancers (SCPC) that are “therapy indifferent” to androgen receptor signaling inhibitors (ARSi).^54,56^ The switch from PC to SCPC is characterized by the presence of neuroendocrine markers (chromogranin A, synaptophysin, and others), and the absence of androgen receptor (AR) and serum prostate-specific antigen.^57^ Functionally, SCPC cells have an elevated metastatic potential, enhanced stemness, heightened therapeutic resistance, and a worse prognosis.^18,58^ Inactivation of *TP53*, *RB1*, *PTEN*, and amplification of *MYCN* have been reported to be involved at the molecular level of the cell identity shift.^18,59^ Mutations in these candidate genes provide a specific genomic background that enables epigenetic regulation towards tumor plasticity.^48,60^

Moreover, epigenetic events are also indispensable. NET of PC cells towards SCPC is associated with the well-established undifferentiated cell marker SRY-Box Transcription Factor 2 (SOX2), epigenetic regulator histone methyltransferase enhancer of zeste homolog 2 (EZH2), the catalytic subunit of the polycomb repressive complex 2 (PRC2), and RE1-silencing transcription factor (REST).^14,18,61–64^ A recent study by Cyrta et al.^65^ suggested that the switch/sucrose non-fermentable (SWI/SNF) chromatin remodeling complexes may also be a potential regulator of SCPC lineage plasticity. Correspondingly, SCPC can be reverted to its original lineage by the re-establishment of *TP53* and *RB1* as well as re-exposure to androgens, or inhibition of SOX2, thus re-sensitized to ARSi treatment.^61,62^ This reversibility of lineage plasticity could be of critical importance as a future target in PC therapy.

Like SCPC, LUAD undergoes NET through LUAD – small-cell lung cancer (SCLC) transition, which enables the tumor to escape from EGFR-TKI treatment and gain a higher level of malignancy.^48,54,66^ Although NET is also identified in non-EGFR-mutant NSCLC, EGFR mutant LUADs are more likely to switch lineage.^67^ Oser et al.^68^ reported that 5–15% of EGFR mutant LUAD patients with TKI resistance showed small cell histology in relapsed tumors, and became sensitive to SCLC chemotherapy.^48^ The mechanism of LUAD NET at the molecular level is similar to that of PC NET. LUADs with loss of *TP53* and *RB1* expression are more likely to transdifferentiate into SCLC tumors, which suggests that these two genes may be critical in promoting transdifferentiation towards a neuroendocrine identity.^60,68–70^ The epigenetic regulator EZH2 is also regarded as a potential therapeutic target to reverse NET.^71,72^

PC and LUAD are currently the most extensively studied cancers exhibiting NET; however, transdifferentiation of epithelial cells is more common. According to a recent study, many epithelial cancers, including bladder, endometrial, and gastric cancer with neuroendocrine gene expression signatures tend to exhibit high-grade tumors and poor prognosis.^66^ This finding indicates that a latent neuroendocrine lineage plasticity may occur in multiple tissues.

### Cancer stem cells

Accumulating evidence suggests that the existence of CSCs within tumor tissues is responsible for tumor initiation and intertumoral heterogeneity. CSCs have the potential to proliferate infinitely, self-renew, and differentiate.^73,74^ A recent study proposed that a CSC is a highly plastic state that results in fast adaptation of tumor cells to varying conditions in the TME.^75^ Previous studies revealed CSCs and adult stem cells share multiple signatures, including the surface markers CD133, CD44, CD24, CD26, CD166, and EPCAM, and intracellular proteins like ALDH1.^76–78^ Additionally, CSCs and stem cells also share similar developmental related signaling pathways, such as WNT, NOTCH, and Hedgehog (Hh).^29,79^

Like normal stem cells, CSCs propagate through asymmetric division, which produces one daughter cell that remains a CSC and one committed progenitor cell that differentiates into a non-CSC.^80,81^ Symmetric division can also occur simultaneously, with the production of either two CSCs (symmetric renewal) or two committed cells (symmetric commitment).^80,82^ The transition between CSC and non-CSC states seems irreversible and dynamically balanced. Under certain circumstances, however, the balance can shift in either direction. A shift towards renewal results in replenishment of the CSC pool and consequently produces a more aggressive, undifferentiated phenotype. A shift towards non-CSCs suggests tumor-formation.^29^ Equilibrium represents the plasticity of tumor cell fate and can be manipulated by various influencing factors. For example, a second mutation introduced in chronic-phase chronic myeloid leukemia (CML) has been found to trigger an imbalance that favors symmetric renewal and results in higher malignancy.^83^ EMT-TF Twist2 has been reported to induce stemness properties in lung CSCs by moving the balance towards symmetric division, while repressing asymmetric division.^84^ Other epigenetic regulations, including deoxyribonucleic acid (DNA) methylation, histone modification, and bivalent chromatin state, have been found to contribute to CSC plasticity.^78,85^ These studies indicate that manipulation of CSC division may be promising for cancer control.

CSCs and EMT share a high degree of consistency and may be a partially overlapping concept.^86,87^ As mentioned, the EMT program largely facilitates tumor cell dissemination. CSCs are also enriched in disseminated tumor cells and feature EMT expression signatures.^88^ Circulating tumor cells express a remarkably congruent transcriptomic profile with CSCs and have been found to overexpress both EMT and stem cell markers.^89^ CSCs led to therapeutic resistance through various mechanisms. For example, CSCs can specifically inherit effective drug-discharging ATP-binding cassette (ABC) transporters from asymmetrical division, which enable tumors to sustain long-term expansion.^90,91^ CSCs can also facilitate efficient DNA damage.^92–94^ Another mechanism of therapeutic resistance in CSCs is the formation of a protective niche, an immunosuppressive microenvironment characterized by low infiltration of immune cells and downregulated signaling by the programmed cell death ligand 1(PD-L1).^79,95^ Non-CSCs also help to maintain of a pool of CSCs by secreting supportive signals such as WNT and brain-derived neurotrophic factor (BDNF), as well as supportive factors such as interleukin (IL)-6 and IL-8.^96–98^

## Mechanism of therapy-induced TCP

Various therapies have been developed to prolong survival and improve the quality of life of cancer patients, yet therapy-induced drug resistance remains a major challenge to achieving the desired clinical outcomes. Notably, up to 90% cancer-associated deaths are attributed to drug resistance.^99^ Acquired drug resistance can be induced by targeted treatments and is associated with TCP, the “hide-and-seek” behavior of cancer cells. The drug-resistant phenotype of cancer cells is not heritable but is a transient and reversible process.^16^ Therefore, a comprehensive understanding of the mechanism of such induced plasticity should greatly facilitate the development of novel therapies. Epigenetic alterations, transcription factors, key signaling pathways, and TME can each potentially contribute to the therapy-induced plasticity in multiple cancers.

### Epigenetic mechanisms

**Fig. 2:**
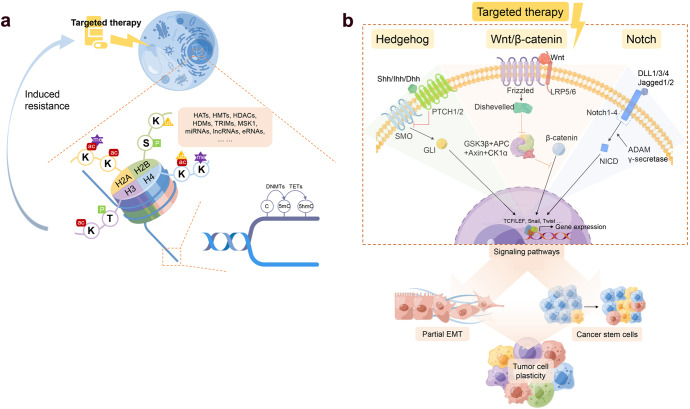
Therapeutic resistance involvement in epigenetic modifications and EMT/MET. **a** Schematic representation of therapy-induced drug resistance through chemical epigenetic modifications. Four core histone proteins (H2A, H2B, H3, and H4) can be diversely modified by multiple enzymes that result in methylation, acetylation, phosphorylation, or ubiquitylation. DNA can be methylated at the 5-carbon of the cytosine base to form 5-methylcytosine (5-mC), which can be further oxidized to form 5-hydroxymethyl cytosine (5-hmC). Enzymes involved in the epigenetic modifications of either histone or DNA can be utilized as potential future therapeutic targets against tumor cell plasticity. K, Lysine; S, Serine; R, Arginine; T, Threonine; me, methylation; ac, acetylation; ub, ubiquitination; P, phosphorylation; HAT, histone acetyltransferase; HMT, histone methyltransferase; HDAC, histone deacetylase; HDM, histone demethylase; TRIM, tripartite mortif; MSK1, mitogen- and stress-activated protein kinase 1; DNMT, DNA methyltransferase; TET, ten-eleven translocation enzymes. **b** Primary tumor cells undergo EMT and MET to achieve metastasis. Primary tumor cells undergo EMT by losing epithelial traits and acquiring mesenchymal characteristics, which enables tumor cells to migrate and invade into blood or lymphatic vessels. In circulation, tumor cells exhibit a “partial EMT” within the epithelial-mesenchymal spectrum. During colonization, migratory tumor cells with mesenchymal traits undergo MET to restore epithelial characteristics and proliferate to form a secondary tumor. Therapeutic resistance and invasiveness are higher in mesenchymal states, whereas a hybrid state of epithelial and mesenchymal indicates the highest level of stemness and capacity of self-adaptation. The process of EMT/MET is regulated by crosstalk among genomic factors, MET-TFs, and multiple signaling pathways. EM, epithelial-mesenchymal transition, MET mesenchymal-epithelial transition, TF transcription factor, miR microRNA

**Fig. 3:**
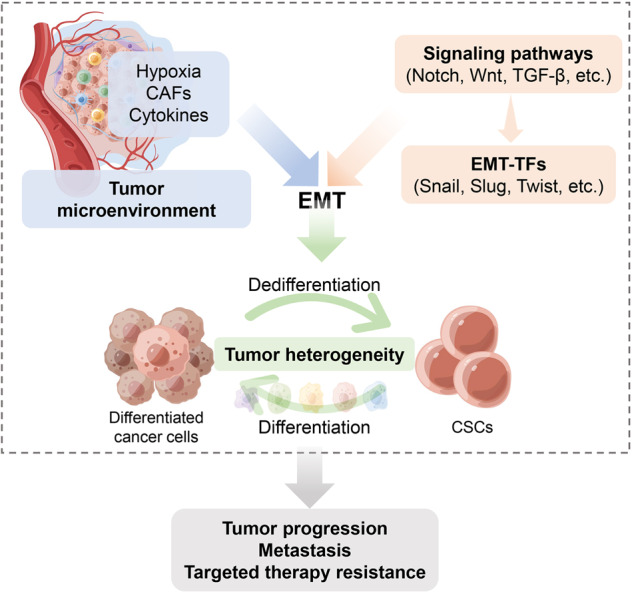
Mechanisms driving tumor cell plasticity and drug resistance. Both tumor microenvironment factors and EMT-related signaling pathways regulate the EMT process. The activation of EMT facilitates the dedifferentiation of non-cancer stem cells (CSCs) to CSC, which contributies to tumor heterogeneity

Therapy-induced epigenetic plasticity largely contributes to the reprogramming of tumor cells and phenotypic switching towards therapy resistance. The epigenetic modifications can be either chemical (DNA methylation and histone modification) or structural (chromatin remodeling and inter/intrachromosomal interactions)^100,101^ (Figs. 2a and 3).

DNA methylation is widespread in the human genome and functions differently based on various methylated regions.^99^ The aberrant methylation status of gene promoters plays an instrumental role in acquired drug resistance in a variety of tumor types.^102,103^ The most common mechanisms of drug resistance include hypomethylation of gene promoters of drug efflux, hypermethylation of promoters of pro-apoptotic genes, and altered methylation levels of DNA-repair genes.^99^ Global loss of DNA methylation and histone acetylation has been observed in therapy-induced drug-resistant tumor cells under extended treatment with the neoadjuvant aromatase inhibitor (letrozole). Such epigenetic alterations result in tumor cells escaping from dormancy and acquiring resistance after targeted therapy in breast cancer patients.^104^

In contrast, indifference or resistance to endocrine therapy in ER-positive breast cancer is due to hypermethylation of estrogen-responsive enhancers.^105^ Moreover, effective therapies targeting the AR in PC inadvertently promote NET. The lineage switching into neuroendocrine PC was associated with increased DNA methylation, turning the original PC into a lethal, castration-resistant type. This process can be reversed by inhibition of DNA methyltransferase (DNMT).^106^ Histone modification, represented by euchromatin histone lysine methyltransferase 2 (EHMT2), has been implicated in acquired drug resistance following targeted therapy. EHMT2 overexpression was observed in erlotinib resistance in NSCLC, leading to transcriptional activation of *PTEN* and repressed Akt signaling, whereas inhibition of EHMT2 reverses the acquired indifference to therapy and re-sensitizes NSCLC cells to EGFR-TKI treatment.^107^ Histone modifications, either via EHMT2 or histone deacetylase 3 (HDAC3), can result in acquired resistance against mitogen-activated extracellular signal-regulated kinase inhibitors (MEKi) treatment in pancreatic ductal adenocarcinoma (PDAC).^108^

Structural epigenetic alterations, including changes in high-order structures of chromatin and interactions among distal regulatory elements, may also be involved in therapy-induced TCP. For example, targeted therapy using γ-secretase inhibitors in T cell acute lymphoblastic leukemias (T-ALL) can induce the expansion of a rare group of pre-existing persister cells. These persisters contain more compact chromatin and higher levels of repressive histone modifications compared to γ-secretase inhibitor-sensitive naïve cells.^109^ Similar to trametinib-resistant triple-negative breast cancer (TNBC), remodeling of enhancers and promoters was observed accompanied with adaptive transcriptomic responses.^110^

### The role of transcription factors in controlling TCP

A network of transcription factors also plays an important role in TCP against targeted therapy. EMT-TFs like Twist and Snail actively respond to targeted therapy during the EMT process and result in enhanced plasticity and mesenchymal switching.^111,112^ In addition to multiple EMT-TFs, several other transcription factors function in controlling TCP.

AR is known as a ligand-activated transcription factor which regulates gene expression in the prostate epithelium.^113^ AR-targeted inhibitors, including bicalutamide and enzalutamide, have been developed for PC treatment.^114,115^ However, upon castration therapy, evading mechanisms are launched by AR amplification, point mutation, splicing variants, as well as substitution of AR functions by glucocorticoid receptors.^116^

Signal transducer and activator of transcription 3 (STAT3) is a transcription factor that has been studied extensively in multiple cancers. Previous studies have suggested that STAT3 activation is associated with trastuzumab as well as trastuzumab-emtansine resistance in human epidermal growth factor receptor 2 (HER2)-positive breast cancer.^117,118^ In triple-negative (ER-/PR-/HER2-) breast cancer (TNBC) patients, a strong transcriptomic response was observed after treatment with trametinib for seven continuous days, with up to 22% of the transcriptome either significantly up- or downregulated.^110^ The super transcription factor cellular MYC underwent rapid degradation during the treatment process and functioned as a controller of genomic reorganization and transcriptional complex recruitment in the phenotypic change of tumors.^110,119^

In melanoma, an assay for transposase-accessible chromatin using sequencing (ATAC-seq) revealed an initial loss of transcription factor binding sites, followed by an increase in accessible sites, during BRAF inhibition (BRAFi) treatment. These results indicated that targeted therapy altered transcription factors.^120^ According to a recent study by Yang et al.,^121^ activating transcription factor 4 (ATF4) stress signaling led to rapid tumor evasion within a few days after the initiation of mitogen-activated protein kinase (MAPK) pathway inhibition, and was accompanied by elevated levels of microphthalmia-associated transcription factor (MITF). This was followed by tumor cell dedifferentiation and a dormant phenotype marked with decrease in MITF expression. Additionally, the helix-loop-helix transcription factor inhibitor of DNA binding 3 (ID3) was upregulated after BRAFi therapy compared to that in the pretreatment state. ID3 acted as a transcriptional repressor of sex-determining region Y-box 10 (SOX10) and MITF, thereby promoting tumor cell transformation to a drug-resistant phenotype.^122^ Moreover, Sun et al.^123^ also observed an increase in the percentage of SOX10^low^ EGFR^high^ melanoma cells after BRAFi and MEKi treatment. This subset of tumor cells displayed a slow-cycling phenotype in response to these targeted drugs, highlighting the potential of transcription factors to mediate plasticity in melanoma.

The regulation patterns of the numerous known transcription factors are quite distinct in targeted therapy-induced tumor cell lines.^120,124^ This complexity indicates that tumors acquire resistance against targeted therapies in a patient- and drug-dependent manner. Hence, the transcription factors and their interaction modes remain to be explored.

### Key signaling pathways in controlling TCP

Numerous signaling pathways are involved in embryonic development, and a re-activation of these signaling pathways is critical in tumor progression and therapy resistance. Interaction of these conserved signaling pathways (e.g., Hh, Wnt, and Notch pathways) and their crosstalk with other oncogenic pathways (e.g., Nuclear factor kappa B (NF-κB), PI3K/AKT/Mammalian target of rapamycin (mTOR), and MAPK pathways) contributes significantly to TCP by regulating the fluctuating expression of downstream effectors^125–128^ (Fig. 2b). Phenotypic switching driven by multiple signaling pathways is a huge challenge to successful tumor treatment.

Alterations of the Hh (Hedgehog) signaling pathway occur in one-third of cancers, and deregulation of the Hh signaling pathway plays an important role in therapeutic resistance.^15,129–132^ Aberrant activation of Hh signaling and subsequent upregulation of downstream effector glioma-associated transcription factor 2 (GLI2) was observed in bortezomib-resistant myeloma cells.^133,134^ Further studies demonstrated that Hh signaling is involved in CSC self-renewal in multiple myeloma, and blocking Hh signaling can lead to CSC differentiation.^135^ In TNBC, the Hh ligand produced by tumor cells can reprogram cancer-associated fibroblasts (CAFs) to promote a CSC-phenotype switching and the acquisition of chemo-resistant traits.^136^ Therefore, an effective Hh signaling pathway blockade is capable of lowering CSC proliferation, metastasis, and the EMT program, thereby controlling TCP.

Wnt signaling pathway is also highly associated with TCP. Wnt signaling is complex and can function through either an autocrine or a paracrine mode, by canonical (Wnt/β-catenin pathway) or non-canonical (the planar cell polarity pathway) pathways. Wnt signaling contributes to EMT regulation and TCP.^137^ In most cases, the Wnt/β-catenin signaling pathway is constitutively activated, which increases the expression of Snail, a regulator of EMT. Inhibition of the Wnt/β-catenin pathway inhibits EMT and tumor metastasis in a colorectal cancer model.^138^ Slug is another EMT modulator that is considered to be a downstream protein in Wnt/β-catenin-mediated tumor invasion. Moon et al. reported that concurrent expression of β-catenin and Slug predicts lymph node metastasis and survival rate in patients with head and neck squamous cell carcinoma.^139^ Overexpression of Slug can induce cancer cell stemness and enhance cell invasion and metastasis. Sun et al. reported that Slug overexpression is associated with the invasiveness of liver cancer both in vitro and in vivo.^140^ In contrast, overexpression of Snail did not have a similar effect in liver cancer cells. The Wnt pathway has been linked to increased expression of another EMT transcription factor, Twist, in mammary epithelial cells.^141,142^

Accumulating data show that TWIST not only induces EMT, but also promotes the formation of CSCs and enhances tumorigenesis.^143^ Therefore, inhibiting these EMT mediators may represent the strategy for inhibiting tumor metastasis and recurrence. Additionally, activation of the Wnt signaling pathway reportedly controls the stemness of CSCs in different types of cancers including hepatocellular carcinoma (HCC), and colon, lung, and other cancers.^144–148^ According to clinical practice, targeted treatment using MEKi (selumetinib, trametinib, and PD318088) inadvertently increased Wnt activity and led to enhanced stemness and tumor relapse in colorectal cancer.^149^ Similarly, valproic acid, which was originally regarded as a histone deacetylase (HDAC) inhibitor in breast cancer treatment, can activate Wnt signaling pathway and promote the pool of mammary CSCs, leading to tumor progression.^150^ Hence, attention should be paid to unexpected phenotypic switching regulated by Wnt signaling pathway in the processes of targeted therapy.

The Notch signaling pathway also enriches CSCs and regulates the EMT program in multiple cancers, including breast and lung cancer, pancreatic ductal adenocarcinoma, and malignant mesothelioma.^151–155^ A previous finding showed a change in EMT status in lung adenocarcinoma upon treatment with gefitinib.^156^ Subsequent studies further confirmed that the EMT process resulted from the activation of Notch-1 signaling pathway and led to gefitinib-acquired resistance.^157^ Notch signaling is one of the master regulators of neuroendocrine transdifferentiation in NSCLC, which enables NSCLC to transdifferentiate into SCLC and acquire resistance to EGFR-TKIs after initial targeted treatment.^154^

By extension, the Notch pathway has been discovered to play a crucial role in the EMT process in prostate cancer cells, promote a transformation towards basal stem-like properties, and lead to a castration-resistant phenotype.^158,159^ Components of the Notch signaling pathway are expressed at significant levels in tumor invasive regions, which suggests its crucial role in EMT regulation. A recent paper by Natsuizaka et al. reported that Notch1 signaling and EMT work closely to promote squamous cell carcinoma tumor initiation. Moreover, another important protein, TGF-β, guides Notch1 to drive EMT.^160^ A study by Zeng et al. showed that inhibition of Notch1 reverses the EMT process and overcomes cisplatin resistance in breast cancer cells.^161^ The data also suggested that high expression of Notch1 predicts a poor prognosis in TNBC patients. Saad et al. demonstrated that the Notch-mediated activation of EMT is associated with the regulation of EMT transcription factors.^162^ The two transcription factors, Snail and Slug, are involved in the inhibition of E-cadherin and β-catenin mediated by the Notch pathway. Inhibition of Notch activation limits the increase of MMP-2 and −9 expression, while it decreases Snail expression. The study provided direct evidence that Notch signaling induces EMT via the Snail pathway. Surprisingly, Notch signaling can synergistically work with other pathways to facilitate the EMT process. The crosstalk between these pathways is mediated by suppressor of mothers against decapentaplegic (SMADs) proteins that associate with other transcription factors for regulating the expression of genes required for the transition to mesenchymal characteristics. A single-cell sequencing study by Deshmukh et al. revealed both sequential and parallel activation of EMT signaling pathways. The Notch pathway also functions as a crucial regulator of TGF-β–induced EMT.^163^

### The role of TME in controlling TCP

TME is a key player in TCP induced by targeted treatment. The characteristics of TME, including immune and stromal cells, cytokines, exosomes, hypoxia, pH change, nutritional deficiency, and angiogenesis, are key regulators of TCP.^164–167^

CAFs are recognized as major contributors in the crosstalk between tumor cells and adjacent stromal cells.^168^ By producing a variety of bioactive molecules such as growth factors and cytokines, CAFs exert a profound impact on TME and influence TCP,^96,169,170^ and have long been described as crucial players in the EMT program. CAFs also participate in CSC pool expansion.^171–173^ The effect of CAFs in manipulating therapy resistance was reported by Apicella et al.^174^ In this study, a prolonged treatment with TKIs generated resistance to erlotinib in vivo, but not in vitro. This intriguing phenomenon was explained by the existence of CAFs. Erlotinib treatment in vivo induces a metabolic rewiring towards aerobic glycolysis and increases lactate production; the secreted lactate triggers the overproduction of hepatocyte growth factor (HGF) in CAFs. Engulfed lactate and the subsequently increased HGF then activate the MET signaling pathway in cancer cells and cause resistance to erlotinib.^175–177^ Moreover, hypoxia-induced glycolysis represses natural killer (NK) cells via lactate accumulation and reduces cytotoxicity by decreased level of NKG2D, CD16, perforin, and granzyme B, thereby forming an immunosuppressive TME. Such hypoxia-related TME conditions were reported to play a role in resistance to therapeutic monoclonal antibodies.^178,179^

Cytokines secreted by immune and stromal cells also provide clues for acquired TCP induced by targeted therapy.^180–182^ According to Li et al.,^183^ IL-6 in EFGR-mutant NSCLC patients was significantly increased upon osimertinib therapy. This associated Upregulation of laminin α5, which is among the most widely distributed laminins and is a major component of extracellular matrix (ECM), also occurred. By upregulation of laminin α5, IL-6 remodeled ECM and activated a downstream target focal adhesion kinase (FAK), thus contributing to acquired resistance against Osimertinib.^183–185^ FAK activation was confirmed to be dispensable for IL-6 production in the maintenance of mesenchymal stem cells and can lead to acquired resistance of gefitinib as well.^186,187^

## Contribution of TCP to targeted therapy resistance

**Fig. 4:**
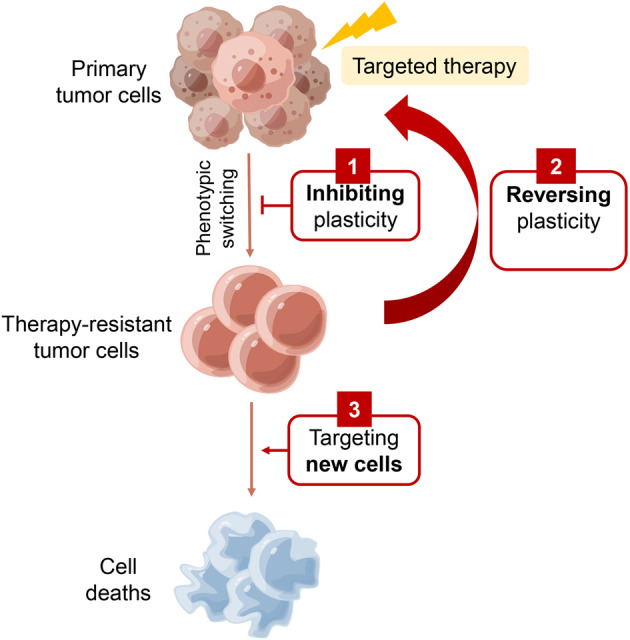
Therapeutic strategies targeting tumor cell plasticity. Three main strategies can be used in combination treatment against tumor cell plasticity: 1) prevent tumor cell plasticity; 2) reverse the phenotypic switching; and 3) direct therapy to target the induced therapy-resistant tumor cells

Targeted therapy is one of the most successful treatments that strike at the heart of a wide range of cancers. Nonetheless, the emergence of drug resistance significantly impairs the efficacy of targeted drugs and contributes to cancer mortality. Drug target mutations and activation of bypass pathways are known to confer resistance in cancer cells to targeted inhibitors. With an increasing understanding of TCP, it is now recognized that TCP is an important factor in mediating drug resistance. In this section, we focus on the role of TCP in shaping tumor cell characteristics and mediating targeted therapy resistance (Fig. 4).

### Epithelial–mesenchymal transition related factors in mediating drug resistance

As described above, EMT plays a critical role in mediating TCP and resistance to targeted therapy. The principal mechanisms of EMT-related drug resistance can be attributed to slow cell proliferation, increased drug efflux, and escape from apoptosis. Lung cancer is by far the most well-documented subtype of cancer that links EMT and targeted drug resistance. Namba et al. demonstrated that the upregulation of EMT-related gene *AXL* contributes to acquired resistance to osimertinib.^188^ Other EMT-related transcription factors, such as Snail, Slug, and ZEB1/2, are also reported to cause TKI resistance by regulation of EMT-related signaling pathways.^189–191^ Therefore, a comprehensive understanding of the EMT process is critical to combat targeted therapy resistance. In this subsection, we delineate the current research on signaling pathways and related factors that modulate EMT associated with drug resistance.

Signaling pathways including Notch, Wnt/β-catenin, and TGF-β play crucial roles in activating EMT-related transcription factors.^192^ Evidence supports that EMT transcription factors are involved in targeted therapy resistance, especially towards EGFR inhibitors. Consequently, signaling pathways that regulate the EMT process are important in mediating drug resistance. Chang et al. reported that Slug contributes to gefitinib resistance in NSCLC cells through downregulation of Bim and inhibition of caspase-9 activity.^193^ Among EMT inducers, TGF-β is significant because of its potency in inducing EMT and its roles in cancer-associated EMT.^194^ TGF-β-induced EMT is essential in cellular activities such as glycolysis and lipid/choline metabolism reprogramming.^195^ In response to TGF-β, cancer cells undergo complete or partial EMT, where epithelial and mesenchymal markers co-exist on cancer cells.^37^ The partial EMT status is characterized by an aggressive phenotype that renders cancer cells with high stemness and cell plasticity.^196^ Upon stimulation of TGF-β receptors, TGF-β signaling is divided into SMAD-dependent and -independent pathways. Both TGF-β/SMAD and non-SMAD pathways play a significant role in mediating EMT and promoting cell plasticity.^197^ Additionally, signaling pathways such as PI3K/AKT, mTOR, and MAPK are reported to play essential roles in TGF-β-induced EMT.^198,199^ Surprisingly, TGF-β-induced EMT can be reversed. A study conducted by Katsuno et al. found that upon TGF-β removal, mesenchymal cells revert to an epithelial cell phenotype.^200^ Moreover, prolonged TGF-β exposure promotes a stable EMT process in cancer cells, which is accompanied by increased tumor stemness and cancer drug resistance. The authors also identified that mTOR inhibitors can be a promising agent to target CSCs by antagonizing the TGF-β-induced EMT process.

MicroRNA (miRNAs) are small non-coding RNAs that play a pivotal role in the regulation of both EMT that promotes tumor metastasis and MET that facilitates metastatic colonization.^201^ EMT-related miRNAs are documented to impact cancer cell stemness and drug resistance. Recent evidence showed that miRNAs may affect the EMT process by targeting related transcription factors, including Snail, Slug, Twist, and ZEB1/2.^202^ The expression level of the Snail transcription factor may be controlled by multiple metastasis-related miRNAs, including miR-153, miR-203, miR-204, and miR-34c.^203^ The miR-30 family are well-documented mediators of EMT transcription factor Snail in different cancer subtypes, including lung and breast cancer.^204^ Liu et al. showed that miR-1 and miR-200 can inhibit EMT and mesenchymal differentiation via Slug-dependent mechanisms.^205^ Other newly identified EMT-related miRNAs include miR-27b-3p,^206^ which promotes the generation of circulating tumor cells, and miRNA-490-3p, which promotes the metastasis of invasive ductal carcinoma.^207^ Nonetheless, recent studies have reported that some miRNAs, such as miRNA-128 MiRNA-155-5p,^208,209^ are able to hinder the EMT process through inhibition of diverse EMT-related signaling pathways. With the increased understanding of miRNAs in EMT and TCP, different strategies focused on hindering miRNA function or miRNA delivery into tumor cells can be utilized to overcome cancer drug resistance.

### Tumor heterogeneity

Tumor heterogeneity is defined as the existence of a subpopulation of cancer cells with different genotypes and phenotypes that exhibit various characteristics within a primary tumor. Heterogeneity may lead to divergent sensitivity to treatment and facilitate the development of drug resistance.^210^

The most recent tumor heterogeneity model is known as the CSC plasticity model.^211^ CSC is a subpopulation of cancer cells with self-renewal capacity to switch between stem and differentiated states. This dynamic transition can be attributed to gene mutations, epigenetic modification, as well as changes in TME. It should be noted that the EMT signaling pathway plays a central role in mediating CSC transition, which promotes cancer metastasis, recurrence, and drug resistance. Morel et al. reported that the induction of EMT can enhance human breast epithelial cell stemness and tumorigenic characteristics,^212^ which has also been confirmed in colorectal, pancreatic, and liver cancers.^213–215^ Quintana et al. demonstrated that most of the phenotypic heterogeneity in melanoma can be explained by the phenotypic plasticity model.^216^ Although they did not identify a marker that can significantly distinguish tumorigenic from non-tumorigenic cells, CD271 + cells were shown to have little tumorigenic activity. However, the results were inconsistent in different studies,^217^ suggesting that the role of cell plasticity in tumor heterogeneity may vary in cancer subtypes. Another study carried out by Charles et al. showed that nitric oxide can enhance the tumorigenic capacity of platelet-derived growth factor (PDGF)-driven glioma cells in vivo; therefore, it reversibly promotes their ability to form tumors.^218^ Nakano et al. reported the dynamic equilibrium between CSCs and non-CSCs in the development of colorectal cancer. Such equilibrium is mainly controlled by EMT-related TGF-β signaling pathway.^219^ One study demonstrated that, in colon cancer cells, the ablation of LGR5 + CSCs allowed the cancer cells to dedifferentiate into CSCs, which highlights the distinct CSC dependency for primary and metastatic tumor growth.^220^

Multiple studies showed that a high level of tumor heterogeneity is associated with poor response to targeted drugs, including osimertinib, rociletinib, and gefitinib.^221–223^ Chabon et al. demonstrated that multi-drug resistance was observed in 46% patients after targeted therapies, suggesting a frequent intra-tumor heterogeneity.^222^ TCP, i.e., phenotype switching between different signaling pathways, can potentially lead to heterogeneity under therapeutic selective pressure. As mentioned above, cell plasticity is essential in multiple crucial biological processes, including embryonic development, wound healing, and tissue regeneration. In tumors, the reactivation of these mechanisms allows tumor cells to switch between non-CSC and CSC-like phenotypes, leading to tumor progression, metastases, and drug resistance.^224^ Easwaran et al. performed a genome-wide analysis and found that silencing abnormal DNA methylation of promoters can decrease changes of plasticity of cell population and help in locking cell clones with abnormal retention of self-renewal capacity.^225^ Therefore, an effective approach to disrupt TCP may represent a promising strategy to tackle tumor heterogeneity and overcome drug resistance.

### Interplay between TCP and TME

A tumor is not only a group of cancer cells, but also a complex interaction between cancer cells and their surrounding environment, known as the tumor microenvironment (TME), which has attracted substantial research and clinical interest in recent years.^226^ TME consists of tumor, immune (macrophages and lymphocytes), and stromal (stromal fibroblasts and endothelial cells) cells, blood vessels, and extracellular matrix (ECM).^227^ Accumulating data show that cancer cells are able to control the function of cellular and non-cellular components through complex signaling pathways.^228^ The cell-cell and cell-ECM interactions force non-malignant cells to acquire new phenotypes that facilitate the formation, progression, and metastasis of tumor cells. In addition, theinteractions of cancer cells with their TME are crucial to stimulate tumor heterogeneity, clonal evolution, and mediate drug resistance.^229^

TCP allows tumor cells to switch phenotypes and survive treatment through adaptive strategies, and the TME is involved in mediating TCP.^165^ For example, both cancer-associated fibroblasts (CAFs) and hypoxia contribute to oxidative stress, which is an important mediator of multiple adaptive strategies. Hypoxia is a hallmark of the TME and strongly linked to malignant progression, poor clinical response, and therapy resistance.^230^ Recent studies have shown that hypoxia may induce phenotypic switching, leading to drug resistance. Terry et al. reported that hypoxic stress induces phenotypic diversity of EMT in lung cancer, and leads to changes in EMT transcription factors such as SNAI1, SNAI2, and ZEB2, which ultimately result in the resistance to cell-mediated cytotoxicity. Paolicchi et al. suggested that tumor hypoxia is a major contributor to drug resistance and a predisposing factor for increased CSC formation and metastases.^231^ Therefore, one promising cancer treatment strategy is to block the proteins related to hypoxia-induced factor (HIF)-1α, EMT, and mitochondria functions, which in turn inhibits the response to hypoxia. In addition, cytokines such as interferon-gamma and tumor necrosis factor-alpha are believed to enhance stromal cell plasticity and contribute to TME.^232^

The interaction between TME and TCP can significantly contribute to the genetic heterogeneity.^233^ Reynolds et al. demonstrated that the number of genetic mutations arising in cells within tumors is much higher than that in the laboratory culture.^234^ Their results provide strong evidence that TME promotes genetic instability during tumor progression. On the other hand, tumor heterogeneity can lead to TME reprogramming and thus give rise to cancer drug resistance.^235^ Taken together, the interplay between TCP and TME serves an important role in mediating tumor progression, heterogeneity, and drug resistance.

## New strategies for targeting TCP

### Preventing initiation of TCP

Strategies that target TCP include intermittent treatments, combination therapies, and targeting the key nodes of phenotypic switching. A study showed that intermittent treatments and off dosing schedules may double the time of melanoma response to vemurafenib.^74^ However, intermittent treatment strategies are usually difficult to design as drug resistance may be complex and come from multiple mechanisms other than TCP.^236,237^ Combination therapies have also been applied to target parallel activation of key signaling pathways such as Hippo/yes-associated protein (YAP), PI3K, and EGFR,^238,239^ resulting in a significant decrease in the number of residual tumor cells. However, combination therapies usually require more optimized drug administration protocols to maximize responses and limit toxicity due to their complexity.

Other strategies to prevent TCP have focused on inhibiting the key nodes of phenotypic switching genes. Histone demethylases, KDM5A/B and KDM6A, were known to play significant roles in DTP survival.^240–242^ KDM inhibitors have been developed; however, they are still under assessment for their specificity and efficacy.^243–246^ DTPs undergo transcriptional adaptation through changing their global chromatin landscape. Another similar strategy that targets the bromodomain and extra terminal domain (BET) *BRD4* gene overcame trametinib resistance in triple-negative breast cancer and was efficient both in vitro and in vivo.^110^ In basal cell carcinoma, the assessment of inhibition or activation of Wnt and Notch signaling pathways are underperforming in clinical trials to treat pathway-dependent malignancies.^247^ Notch-activated tumor cells tend to undergo apoptosis upon vismodegib treatment. Inhibitions of Retinoid X receptor (RXR) pathways using HX531 together with MAPK inhibitors can significantly delay development of resistance to MAPK inhibitors, compared to treatment with MAPK inhibitors alone.^236^ Discovery of more drug targets that prevent tumor plasticity will enable the design of treatment strategies.

### Targeting the fate of new cells

In addition to preventing the formation of cell plasticity, targeting the emerging drug-resistant new cell identity holds great potential for overcoming tumor plasticity (Fig. 4). Transformation from the NSCLC to SCLC usually causes strong drug resistance due to cell plasticity.^248^ SCLC cells often lose EGFR expression; therefore, they are resistant to EGFR inhibitors, in contrast to the nontransformed NSCLC (specifically LUAD). Transformed SCLCs were more responsive to platinum-etoposide, similar to *de novo* SCLC.^67^ However, compared to *de novo* SCLC, transformed SCLC showed a higher response rate to taxanes yet were resistant to checkpoint inhibitor therapy, which was similar to EGFR-mutant LUAD.^249^ Recently, molecular studies showed that the growth of some mesenchymal cancer cells depends on the expression of phospholipid glutathione peroxidase 4 (GPX4), and inhibition of GPX4 can profoundly decrease DTP, thus preventing tumor relapse.^250^ However, current GPX4 inhibitors are chloroacetamide-containing compounds (such as RLS3), which have been identified to possess only poor pharmacokinetic properties, such as low stability, promiscuity, and bioavailability. The GPX-knockout mouse was lethal, indicating that choosing the therapeutic window that targets this protein is challenging.^251–253^ Thus, finding drugs that target the new cell identity is necessary for designing therapeutic strategies.

### Reversing TCP

TCP can be driven by an inflamed and suppressive TME. Drugs and therapies have been developed against intracellular signaling kinases and extracellular signaling molecules in TME.^254,255^ Additionally, since epigenetic changes contribute to TCP, targeting those processes could potentially re-sensitize tumor cells to drug treatments.

#### IL-8

IL-8, a pro-inflammatory cytokine, is known to promote tumor cell remodeling and is involved in regulation of tumor cell stemness, EMT, and therapy resistance in multiple types of tumors.^256,257^ IL-8 production promotes tumor cells to transform to a mesenchymal phenotype and increase tumor cell migration, leading to metastases.^258^ C-X-C chemokine receptor 1 (CXCR1) antagonist therapies have been found to reverse the IL-8 related events.^259^ Resistance to PI3K/Akt/mTOR pathway inhibition in hepatocellular carcinoma has been associated with both liver cancer stem cells and a high level of IL-8 expression. Rapamycin, the mTOR inhibitor, was able to decrease the IL-8 expression, thus decrease the resistance to sorafenib.^260^ A neutralizing antibody, HuMAX-IL8, was used to deplete IL-8 secretion and treat human TNBC.^261^ Another study showed that neutralizing IL-8 can alleviate resistance to erlotinib in NSCLC, thereby enhancing sensitivity in chemotherapy-resistant cells.^262^ Inhibition of CXCR2 with SB225002 also alleviated the IL-8-related resistance in sorafenib-resistant ovarian tumor cells.^263^

#### TGF-β

Reversing EMT by blockage of TGF-β signaling is acknowledged as a promising way to reverse TCP, as TGF-β has been widely recognized as the major inducer of EMT.^264^ A recent single-cell transcriptome-based study showed that blocking TGF-β1 could restore stromal plasticity in tumors by affecting a subset of interferon-licensed fibroblasts.^265^ However, the pleiotropic functions of TGF-β1 in cancer render therapeutic inhibition of this pathway challenging.^266^ TGF-β1 inhibition in various combination therapies that target the drug-induced plasticity is under evaluation.^267^ Galunisertib (LY2157299 monohydrate), a small molecule inhibitor that inhibits TGFβRI ALK5 kinase, has been used in numerous preclinical trials.^268^ Forskolin and cholera toxin have been identified through a drug screen for E-cadherin promoter activation.^269^ JMF3086, a dual inhibitor that targets both HDAC and 3-hydroxy-3-methylglutaryl coenzyme A reductase, can restore the sensitivity of NSCLC to EGFR-TKIs (34). A preclinical study showed that galunisertib has the potential to regulate pancreatic cancer cell growth through prevention of TGF-β mediated E-cadherin downregulation.^270^ Galunisertib also demonstrated anti-tumor efficacy in patients with pancreatic cancer as well as in a subset of patients with advanced HCC.^271^ A recent study also demonstrated that inhibition of TGF-β and leukocyte-associated immunoglobin like receptor-1 (LAIR-1) can remodel TME, thereby enabling programmed-death ligand 1 (PD-L1)-mediated tumor eradication.^272^ Dual-blockade of CD73-TGFβ promotes a multifaceted inflammatory environment in TNBC, by diminishing myeloid-derived suppressor cells and M2-macrophages but increasing cytotoxic T cells and activated dendritic cells.^273^ These studies and novel drugs have provided strong evidence that blocking TGF-β can reverse TCP, thereby sensitizing tumor cells to targeted or immune-mediated cell death.

#### EZH2

EZH2, the catalytic subunit of polycomb repressive complex 2 (PRC2), is the central player in the process of epigenetic gene silencing. EZH2 promotes tumor cell survival, proliferation, EMT, and invasion, as well as drug resistance.^274^ Reversible transition between EMT and MET are key aspects of TCP. Interestingly, EZH2 was recently found to support TCP through MET facilitation and tumor colonization.^275^ Overexpression of EZH2 was associated with the conversion of prostate adenocarcinoma to neuroendocrine prostate cancer (NEPC), which is a more aggressive variant.^61,276^ Inhibition of EZH2 can reverse the cell lineage switch, leading to a decrease in neuroendocrine related key factors and restoring the sensitivity of NEPC cells to enzalutamide. Some EZH2 inhibitors (for example, CPI-1205 and Tazemetostat) have entered phase I and II clinical trials for lymphomas and solid tumors.^277,278^

#### Other targets and pathways

Other targets and pathways with potential ability to reverse TCP are emerging. Ferroptosis was identified as a new type of programmed cell death that can be harnessed to target drug resistance and EMT. Gagliardi et al. identified that EMT-associated gene expression reprogramming process that determines ferroptotic susceptibility cannot be applied to metastatic-derived cells, but SCL7A11 was a valuable marker to predict the susceptibility.^279^ Increased sensitivity to ferroptosis through crosstalk of *BACH1* target genes can also impose programmed vulnerability upon cancer cells.^280^ Other than ferroptosis, HDAC inhibition was recently found to reverse EBV-induced dedifferentiation in nasopharyngeal carcinoma.^281^ The Yap-Sox9 axis was recently reported to determine hepatocyte plasticity in hepatocarcinogenesis and could be a promising target.^282^ Some other SOX factors have also been considered as promising candidates through proteolysis-targeting chimeric molecules or thalidomide analogues.^283,284^ An anti-CLDN6 antibody-drug conjugate has been reported to target tumor lineage plasticity.^285^ Another recent study showed that blocking the MNK1/2-eIF4E axis could inhibit melanoma phenotypic switching, thereby, sensitizing melanoma to anti–PD-1 immunotherapy.^286^ These studies suggest that reversing TCP through other targets could be a propitious future direction.

## New therapies for overcoming TCP-induced drug resistance

**Fig. 5:**
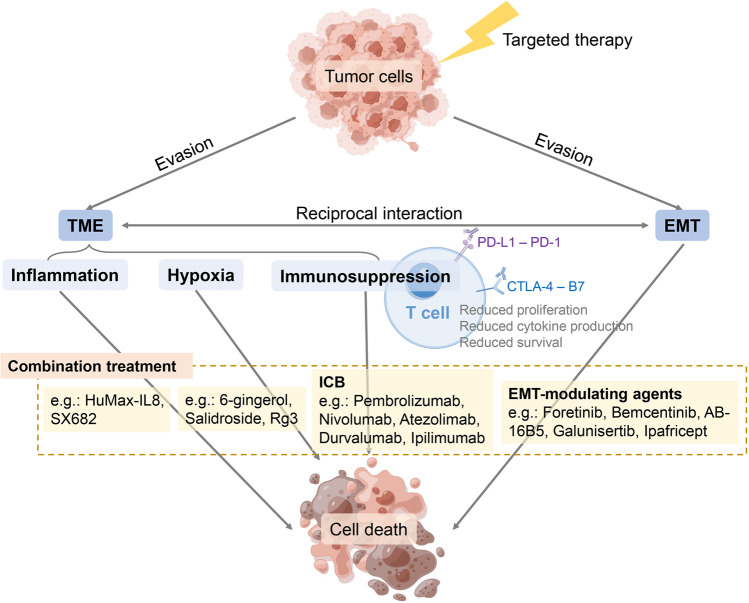
Combination treatments for overcoming the tumor cell plasticity induced-therapy resistance. Tumor cells evade targeted treatments by interacting with TME and via EMT programs. Inflammation, hypoxia, and immunosuppressive TME contribute to therapy evasion, and such induced-resistance could be inhibited by a combination treatment including anti-inflammation and anti-hypoxic drugs and immunosuppressive ICB regimens. EMT-modulating agents, on the other hand, could also deal with plasticity induced-therapy resistance as a combination treatment and lead to tumor cell death. TME tumor microenvironment, EMT epithelial-to-mesenchymal transition, ICB immune checkpoint blockade, Rg3 ginseng-extracted 20(R)-Ginsenoside

TCP enables tumor cells to evade traditional anti-tumor therapies. Therefore, agents that block these escape routes can deprive the residual tumor cells of their hiding place and ensure positive treatment results (Fig. 5).

### With immune checkpoint blockade (ICB)

An immunosuppressive TME is formed upon drug exposure as a consequence of the interaction between non-tumor and tumor cells. It is worth mentioning that the expression of immune checkpoint proteins, including programmed cell death 1 receptor (PD-1) and (PD-L1), cytotoxic T lymphocyte-associated protein 4 (CTLA-4), T cell immunoglobulin 3 (TIM-3), lymphocyte activation gene 3 (LAG-3), and T cell immunoreceptor with Ig and ITIM domains (TIGIT) contribute significantly to immunosuppression and serve as a bridge between EMT programs and immunosuppression.^287–291^ A positive correlation between EMT and immune checkpoint proteins was identified in multiple cancer types, including lung, breast, colorectum, oral, and esophageal cancers,^289,292–297^ and a higher expression of these checkpoint molecules indicates worse prognosis in a broad range of cancers.^298–300^ The functional relationship between immune checkpoint proteins and therapy evasion suggests that a combination treatment with immune checkpoint blockade (ICB) could be promising in developing a new approach against TCP.

Combining targeted therapy with ICB seems feasible according to current practice. Traditional EGFR-TKI was found to interfere with TME, thus laying a theoretical foundation for ICB application in EGFR-TKI-resistant NSCLC, especially in those with upregulated PD-L1 expression.^301–303^ Moreover, recent studies revealed that dynamic changes occur in TME in response to EGFR-TKIs and suggested that a combination treatment with immune-mediated anticancer approaches could be beneficial.^302,304–307^ EGFR-TKI-ICB combination treatment provided better clinical benefit for NSCLC patients with increased overall response rate (ORR) (41.7% vs 14.3%) and median progression free survival (PFS) (19.5 months vs 1.4 months) in comparison to monotherapy of either pembrolizumab or gefitinib alone.^306^ Patients with short EGFR-TKI-PFS may even benefit more from a combination therapy as a second-line treatment.^308^ A similar combination strategy was applied in multiple cancer types as a so-called basket trial design.^309^ A combination of ICB with antiangiogenic molecular targeted therapy is an attractive approach for treating HCC. According to the IMbrave150 trial, atezolizumab in combination with bevacizumab showed greater benefits compared with sorafenib in advanced HCC patients and suggested that this combination strategy could be used as the first-line standard of care.^310–313^ Additionally, a combination of ICB with other targeted therapeutic approaches, including ATR kinase inhibition and androgen receptor blockade resulted in synergistic therapeutic response in prostate cancer.^314–316^

Combining chemotherapy with ICB can be used to extend a synergistic therapeutic effect.^317,318^ A pooled analysis of three randomized clinical trials indicated that among patients with PD-L1-negative advanced / metastatic NSCLC, efficacy is better with pembrolizumab plus chemotherapy compared with chemotherapy alone.^319^ Similar results were obtained in PD-L1-positive NSCLC patients. As indicated by Zhang et al.,^320^ the combination regimen of toripalimab and pemetrexed/carboplatin was superior to chemotherapy alone. Moreover, an IMpower150 trial showed that a combination of atezolizumab, bevacizumab, carboplatin, and paclitaxel (ABCP) led to significant improvement in median overall survival (OS) in chemotherapy-naïve patients with NSCLC.^321^ Anti-CTLA-4 ipilimumab was also tested in the treatment for NSCLC in combination with nivolumab and platinum doublet chemotherapy and exhibited clinical value as a first-line treatment.^322^ Another phase III clinical trial compared the efficacy of chemotherapy with nivolumab and chemotherapy alone in treatment for advanced gastric, gastroesophageal junction (GEJ), and esophageal adenocarcinoma, and found that the combination strategy had a benefit of significantly superior OS and PFS.^322^ Janjigian et al.^322^ proposed that this combination treatment represented a new first-line therapy for patients with upper gastrointestinal tumors. A recent phase III study (KEYNOTE-811) also confirmed that adding pembrolizumab to standard first-line treatment (trastuzumab plus chemotherapy) in advanced gastric or GEJ adenocarcinoma could markedly improve prognosis.^323^

Though the basket trial design has greatly improved the clinical outcome of multiple cancer types, our understanding of the underlying mechanisms of the combination regimens remains limited. For example, nab-paclitaxel combined with atezolizumab are able to effectively prolong PFS in patients with metastatic TNBC as shown by the IMPASSION130 trial; however, the combination of paclitaxel with atezolizumab failed in the IMPASSION131 trial.^324,325^ Single-cell analyses revealed that baseline CXCL13^+^ T cell largely determined the clinical response in TNBC treatment.^326^ Further studies are warranted to explore the exact mechanisms of different combination strategies to be able to pin-point patients with higher therapeutic potential. Furthermore, novel combination methods are emerging. For example, a bioengineered platelet was designed that combines an inner-loaded doxorubicin and outer-anchored anti-PDL1-crosslinked nanogels. This composite design was reported to have the potential to reduce the recurrence and metastasis rate of postsurgical tumors.^327^

Despite some optimistic reports, the safety profile of combination therapies with ICB remains poorly understood and controversial. In NSCLC treatment, an increased incidence of interstitial lung disease-like events was observed in the combination treatment of osimertinib and durvalumab in the CAURAL (38%) and TATTON (22%) trial.^328–333^ Severe hepatotoxicity was observed in another clinical trial (CheckMate370) investigating the combined effect of nivolumab and crizotininb.^306^ Similar hepatic and immune-related adverse events were also observed in combined treatment with ICBs in melanoma.^334–336^

These described studies imply that a combination with ICB can improve therapeutic outcomes for therapy-resistant patients compared with either therapy alone. The clinical feasibility of the combined strategy warrants further exploration, and the safety profile should be carefully documented. Table 1 provides a list of some relevant clinical trials evaluating the combination therapy with ICB mentioned above.

**Table 1:** Relevant clinical trials of combination treatment with ICB

Clinical trial (NCT identifier)	Conditions	Treatment	Patients	*n*	Phase	States
NCT02039674 (KEYNOTE-021)	NSCLC	Pembrolizumab + Ipilimumab + Erlotinib + Gefitinib + Paclitaxel + Carboplatin + Bevacizumab + Pemetrexed	Patients with unresectable or metastatic NSCLC	267	I/II	Completed
NCT02143466 (TATTON)	NSCLC	Durvalumab + Osimertinib + Selumetinib + Savolitinib	NSCLC patients with progression after EGFR-TKIs, T790M positive	344	I	Active, not recruiting
NCT02366143 (IMpower150)	NSCLC	Atezolizumab + Bevacizumab + Paclitaxel + Carboplatin	Chemotherapy-naïve patients with stage IV non-squamous NSCLC	1202	III	Completed
NCT02454933 (CAURAL)	NSCLC	Durvalumab + Osimertinib	NSCLC patients with progression after EGFR-TKIs, T790M positive	344	I	Active, not recruiting
NCT02574078 (CheckMate 370)	NSCLC	Nivolumab + Erlotinib + Crizotinib + Bevacizumab + Pemetrexed + Nab-Paclitaxel + Paclitaxel + Docetaxel + Gemcitabine + Carboplatin	Patients with advanced NSCLC	341	I/II	Completed
NCT02578680 (KEYNOTE-189)	NSCLC	Pembrolizumab + Cisplatin + Carboplatin + Pemetrexed + Dexamethasone	Patients with metastatic non-squamous NSCLC	616	III	Active, not recruiting
NCT02659059 (CheckMate 568)	NSCLC	Nivolumab + Ipilimumab + Platinum doublet chemotherapy	Stage IV NSCLC	324	II	Active, not recruiting
NCT02775435 (KEYNOTE-407)	NSCLC	Pembrolizumab + Paclitaxel+ Nab-paclitaxel + Carboplatin	Patients with metastatic non-squamous NSCLC	559	III	Active, not recruiting
NCT02872116 (CheckMate649)	Gastric cancer GEJ cancer	Nivolumab + Ipilimumab + Oxaliplatin + Capecitabine + Leucovorin + Fluorouracil	Patients with previously untreated advanced or metastatic gastric or GEJ cancer	2031	III	Active, not recruiting
NCT03434379 (IMbrave150)	HCC	Atezolizumab + Bevacizumab + Sorafenib	Patients with locally advanced or metastatic HCC with no prior systemic treatment	558	III	Active, not recruiting
NCT03615326 (MK-3475-811/KEYNOTE-811)	Gastric neoplasms GEJ adenocarcinoma	Pembrolizumab + Cisplatin + 5-FU + Oxaliplatin + Capecitabine + S-1 + Trastuzumab	HER2 + patients with advanced gastric or GEJ adenocarcinoma	732	III	Active, not recruiting
NCT03924050	NSCLC	Toripalimab + standard chemotherapy	Advanced NSCLC patients with TKI-resistant EGFR-mutated tumors	350	III	Unknown

*NSCLC* nonsmall cell lung cancer, *EGFR-TKI* epidermal growth factor receptor – tyrosine kinase inhibitor, *HCC* hepatocellular carcinoma, *GEJ* gastroesophageal junction, *5-FU* 5-Fluorouracil, *S-1*, tegafur + 5-chloro-2,4-dihydroxypyridine + potassium oxonate, *HER-2* human epidermal growth factor receptor 2

All data are from ClinicalTrials.gov, accessed May 2022

### With EMT-modulating agents

Because EMT plays an essential role in TCP and drug resistance, EMT-targeting compounds hold great potential as novel drugs in reversing drug resistance. However, investigation in this field is limited.

EMT-TFs play an essential role in TCP, and drugs directly targeting these transcription factors might have the potential to halt or reverse the plasticity and become a novel therapeutic approach to repress drug resistance. However, due to the redundancy of overlapping pathways and multitude of factors, it is hard to target and control TCP effectively. Direct targeting of these EMT-TFs is very challenging and there is not yet known EMT-TF-targeting molecule.^19^ Nevertheless, some related molecules involved in redundant EMT pathways have been investigated for clinical use. As a downstream target of YAP and EMT-TF Snail2, anexelekto (AXL) represents an ideal focus of EMT control.^337,338^ AXL-targeting inhibitors (SGI-7079 / Foretinib [XL-880] / Bemcentinib [BGB324] / 20G7-D9 / MYD1-72) have been reported to have an outstanding synergistic effect with erlotinib in NSCLC^40,339,340^ and advanced pancreatic and ovarian cancers,^337^ BRAF inhibitors in melanoma,^341^ and temozolomide with radiation in glioblastoma.^342^ Multitarget AXL inhibitors like SGI-7079 and sunitinib have also shown prominent clinical value in reversing therapeutic resistance.^40,343,344^ Moreover, secreted clusterin, an EMT-related protein which was shown to be significantly upregulated in EMT progression and HCC migration, is a candidate target for HCC therapy.^345–347^ An antibody against secreted clusterin, termed as AB-16B5, was assessed in a phase II clinical trial in combination with docetaxel for the treatment of metastatic NSCLC (Table 2).

**Table 2:** Ongoing clinical trials of combination treatment with EMT-modulating agents

Clinical trial (NCT identifier)	Conditions	Treatments	Patients	n	Phase	States
NCT02922777	NSCLC	Bemcentinib + Docetaxel	Previously treated patients with advanced NSCLC	23	I	Active, not recruiting
NCT02488408	AML	Bemcentinib + Cytarabine + Decitabine	Patients with AML	121	I/II	Active, not recruiting
NCT03184571	NSCLC	Bemcentinib + Pembrolizumab	Previously treated patients with advanced NSCLC	106	II	Recruiting
NCT02872259	Melanoma	Bemcentinib + Dabrafenib + Trametinib	Patients with advanced stage IIIc / IV) melanoma	92	I/II	Recruiting
NCT04364620	NSCLC	AB-16B5 + Docetaxel	Previously treated patients with metastatic NSCLC	40	II	Recruiting
NCT02452008	Prostate Cancer	Galunisertib + Enzalutamide	Patients with metastatic castration-resistant prostate cancer	60	II	Recruiting
NCT02672475	Breast cancer	Galunisertib + Paclitaxel	Patients with metastatic androgen receptor negative or triple negative breast cancer	29	I	Active, not recruiting
NCT01682187	Nasopharyngeal carcinoma	Galunisertib + Gemcitabine + Cisplantin + PD-1 blocking antibody + Radiation	Patients with high-risk locoregionally advanced nasopharyngeal carcinoma	206	II	Not yet recruiting
NCT03206177	Ovarian / Uterine carcinosarcoma	Galunisertib + Paclitaxel/Carboplatin	Patients with newly diagnosed, persistent or recurrent carcinosarcoma of the uterus or ovary	26	I	Active, not recruiting
NCT02688712	Rectal adenocarcinoma	Galunisertib + Capecitabine + Fluorouracil + Surgery + Radiation	Patients with locally advanced rectal adenocarcinoma	50	II	Active, not recruiting
NCT01582269	Glioblastoma	Galunisertib + Lomustine	Patients with glioblastoma	180	II	Active, not recruiting
NCT04396886	Nasopharyngeal carcinoma	Bintrafusp alfa (α-PDL1/TGF-βRII fusion protein)	Previously treated patients with recurrent and metastatic non-keratinizing nasopharyngeal carcinoma	37	II	Recruiting
NCT05005429	Mesothelioma; lung	Bintrafusp alfa (α-PDL1/TGF-βRII fusion protein)	Previously treated advanced malignant pleural mesothelioma	47	II	Recruiting
NCT04349280	Urothelial cancer	Bintrafusp alfa (α-PDL1/TGF-βRII fusion protein)	Metastatic or locally advanced urothelial cancer	25	I	Active, not recruiting
NCT04727541	Biliary tract cancer; Cholangiocarcinoma	Bintrafusp alfa (α-PDL1/TGF-βRII fusion protein)	Patients with resectable biliary tract cancer	24	II	Recruiting
NCT04396535	NSCLC	Bintrafusp alfa (α-PDL1/TGF-βRII fusion protein)	Patients with advanced NSCLC	80	II	Active, not recruiting
NCT05145569	Ovarian cancer	Bintrafusp alfa (α-PDL1/TGF-βRII fusion protein)	Patients with metastatic advanced stage ovarian cancer	33	I	Not yet recruiting
NCT04489940	Breast cancer	Bintrafusp alfa (α-PDL1/TGF-βRII fusion protein)	Patients with HMGA2 expressing triple negative breast cancer	11	II	Active, not recruiting
NCT02521844	Solid tumors	ETC-1922159 + Pembrolizumab	Patients with advanced solid tumors	89	I	Recruiting

*NSCLC* non-small cell lung cancer, *AML* acute myeloid leukemia, *HMGA2* high mobility group AT-Hook 2

All data are from ClinicalTrials.gov, accessed May 2022

Inhibition of EMT-related pathways is a theoretically feasible approach to control TCP. TGF-β, a widely accepted key promoter of EMT and sustainer of mesenchymal and CSC states, contributes to the induction of immunosuppressive TME via hijacking Tregs and NK cells.^44,45,348^ A small molecule inhibitor of TGF-β receptor 1 named galunisertib (LY2157299 monohydrate) is attracting attention. One preclinical evaluation of galunisertib showed an inhibition of TGF-β-mediated downregulation of E-cadherin in mouse pancreatic cancer cell line, indicating an anti-EMT effect.^349^ A recent study by Song et al.^350^ demonstrated a synergistic effect in castration resistant PC using enzalutamide combined with galunisertib. The combinatorial galunisertib treatment successfully sensitized PC cells to traditional androgen deprivation therapy (ADT) and provided a novel therapeutic strategy for control of lineage transdifferentiation. Other studies tested the efficacy of galunisertib in HCC and indicated a prolonged OS using a combination of galunisertib and sorafenib.^351^ Melisi et al.^271^ stated in a phase Ib/II clinical trial that a galunisertib-gemcitabine combination therapy could significantly improve OS in patients with unresectable pancreatic cancer compared with monotherapy with gemcitabine. The same result was found in myelodysplastic syndromes (MDS); galunsertib treatment was associated with hematologic improvement in patients with signs of blockage of early stem cell differentiation. However, some other clinical trials failed to prove the efficacy of galunisertib in HCC, metastatic pancreatic cancer, or glioma.^352–354^ Though such novel drugs can manipulate TCP and potentially re-sensitize tumor cells to immune-mediated cell death, further clinical trials are needed to validate their efficacy in different types of tumors. The ongoing clinical trials investigating the combination treatment with galunisertib are shown in Table 2.

Additionally, a combined immunotherapy of ICB and TGF-β blockade is also promising. In a murine model of metastatic intestinal tumor, the combination of anti-TGF-β and anti-PD-L1 showed an impressive antitumor response compared to either monotherapy.^355^ Currently, new approaches for simultaneous inhibition of TGF-β and PD-L1 are under evaluation. Bintrafusp alfa (also known as M7824) is a recently developed anti-PD-L1/ TGF-βRII fusion protein, which showed superior inhibition of tumor growth in multiple murine models.^356,357^ Other fusion drugs such as anti-CTLA4-TGF-βRII also showed promising efficacy in stimulating antitumor responses.^358^ Several early-phase trials showed encouraging clinical efficacy and manageable safety in different types of cancers.^359–361^ Several ongoing clinical trials are testing the efficacy and safety of bintrafusp alfa (Table 2).

Wnt inhibitors including ETC-1922159 and Ipafricept (also known as OMP-54F28) were also employed in clinical control of various types of solid tumors.^362–365^ Ipafricept combined with chemotherapy showed reasonable tolerance in early-phase clinical trials, and remains a therapeutic target of interest.^363,364,366^ ETC-1922159 is now under assessment in an ongoing clinical trial to test its efficacy as a pembrolizumab combination (Table 2).

### With other therapy

The close interaction between inflammatory TME and EMT is well established and is regarded as a promising target for therapeutic use.^15^ Since TCP drives a highly inflamed TME, neutralizing monoclonal antibodies targeting IL-8 were developed to reverse TCP. HuMax-IL8 (also known as BMS-986253), a neutralizing anti-IL8 antibody that was designed to sequester IL-8 signaling, successfully inhibited the mesenchymal transition in human TNBC cell lines in vitro. Blockade of IL-8 enhanced the tumor cell-killing effect mediated by NK and T cells. Another in vivo study in NSCLC found that IL-8 neutralizing antibodies reversed the tumor plasticity and sensitized tumor cells to erlotinib. Currently, multiple ongoing clinical trials in phase I/II are examining the safety and efficacy of HuMax-IL8 as a combination therapy in various cancer types, including prostate and pancreatic cancer, HCC, NSCLC, and other solid tumors (Table 3). SX-682, another novel drug targeting the inflammatory EMT, was proposed to prevent polymorphonuclear (PMN)-MDSC migration and cooperate well with ICB treatment.^367,368^ Early-phase clinical trials of SX-682 in combination with ICBs (e.g., pembrolizumab and nivolumab) are currently recruiting participants (Table 3).

**Table 3:** Ongoing clinical trials of combination treatment with other therapy

Clinical trial (NCT identifier)	Conditions	Treatment	Patients	n	Phase	States
NCT04848116	SCCHN	HuMax-IL8 + Nivolumab	SCCHN patients who will undergo surgery.	24	II	Recruiting
NCT03689699 (MAGIC-8)	Prostate cancer	HuMax-IL8 + Nivolumab + ADT	Patients with prostatic adenocarcinoma	60	Ib/II	Active, not recruiting
NCT02451982	Pancreatic Cancer	HuMax-IL8 + Nivolumab	Patients with resectable pancreatic adenocarcinoma	76	II	Recruiting
NCT04050462	HCC	HuMax-IL8 + Nivolumab	Advanced HCC patients	74	II	Recruiting
NCT03400332	Advanced cancers	HuMax-IL8 + Nivolumab + Ipilimumab	Patients with advanced solid tumor	372	I/II	Recruiting
NCT04123379	NSCLC or HCC	HuMax-IL8 + Nivolumab	NSCLC or HCC patients	50	II	Recruiting
NCT04572451	Unresectable Solid Tumors	HuMax-IL8 + Nivolumab + SBRT	Patients with advanced / metastatic / unresectable solid tumors progressed on standard therapies	50	I	Recruiting
NCT03161431	Melanoma	SX-682 + Pembrolizumab	Patients with metastatic melanoma	77	I	Recruiting
NCT04599140 (STOPTRAFFIC-1)	Colorectal cancer	SX-682 + Nivolumab	RAS-mutated, microsatellite stable, unresectable or metastatic Colorectal Cancer	53	I/II	Recruiting
NCT04477343	Pancreatic cancer	SX-682 + Nivolumab	Patients with metastatic pancreatic ductal adenocarcinoma	20	I	Recruiting

*SCCHN* Head and neck squamous cell carcinoma, *ADT* androgen deprivation therapy, *HCC* hepatocellular carcinoma, *NSCLC* non-small cell lung cancer, *SBRT* stereotactic body radiotherapy

All data are from ClinicalTrials.gov, accessed May 2022

Hypoxia signaling contributes significantly to EMT and subsequent therapy resistance. Hypoxia-induced factor (HIF), a transcriptional regulator, was reported to be recruited to the hypoxia-responsive element of several EMT-related genes, including Twist, Snail, Vimentin, and TGF-β.^369^ HIF-1α promoted EMT via multiple signaling pathways, including PI3K/Akt, Notch, Wnt, and NF-κB.^345,370^ Treatments targeting hypoxia pathway are deemed as promising blockades against TCP. For example, by blocking the nuclear translocation of HIF-1α, 6-gingerol successfully inhibits the EMT pathway and suppresses proliferation and metastasis of lung cancer.^369^ Salidroside enhances antitumor efficacy of platinum drugs in HCC by promoting the degradation of HIF-1α, thereby inhibiting the EMT of HCC cells.^371^ Similarly in lung cancer, by inhibiting hypoxia-activated NF-κB signaling pathway and EMT, a ginseng-extracted 20(*R*)-Ginsenoside (Rg3) increases the susceptibility of cancer cells to cisplatin treatment.^345^

## Conclusion and perspectives

Here, we present a comprehensive description of TCP development and cancer cell evasion from targeted therapy. We delineate the non-genetic mechanisms of targeted drug-induced TCP in various types of tumors and provide insights into TCP contribution to acquired drug resistance. Owing to the rapid development of experimental techniques, dynamic changes of tumor cells in response to treatments can be observed in high resolution images. The ceaseless change of tumor cells indicates that living things even as simple as single cells show amazing adaptability under ambient pressure. Continuous monitoring of phenotypic switching can be of great help in designing specific dynamic therapeutic regimens and developing such monitoring techniques is worth exploring. We have been able to look into some pathways, like EMT, CSC, and transdifferentiation, that facilitate transformation of tumor cells; however, we still cannot visualize all the mechanisms contributing to TCP.

Research and development for new treatment is never-ending. As an old saying goes, a coin has two sides. In spite of TCP-related therapy escape, TCP also allows therapeutic reprogramming and possibly reversal of tissue attrition. In this review, we summarize new therapeutic strategies that aim to inhibit or reverse TCP and discuss related combination treatment regimens. We note that regulation of plasticity-related TME with ICB or direct imposition of EMT-modulating agents will not bring revolutionary improvement in cancer treatment. However, each small step forward against cancer is worth trying, and the concept of TCP remains valuable for further development of novel therapeutic strategies. Inhibition of unexpected TCP is theoretically feasible by holding the tumor cell unchanged. On the other hand, effective manipulation of TCP also holds great potential, and converting diverse tumor cells into a unified and susceptible state could be an alternative. The basket trial design should be continued to probe new treatment opportunities for different types of cancers, and special attention should be given to searching for new targets to better manipulate TCP.

As for future research on TCP, modern technologies could be of great help. For example, omics technique and capacity of dealing with “big data” has largely broadened our knowledge of the molecular landscape of tumor tissues. Recently developed technologies could enable a simultaneous acquisition of multiple omics data at a single-cell level, and several analytic models have been established to interpret the high-dimensional multimodal data.^372,373^ Single-cell level multi-omics could precisely trace the therapy-induced responses of each cell in heterogeneous tumors from the aspects of transcriptomics, proteomics, epigenomics, even in combination with their spatial distribution. The innovative analytical modalities provide deep and comprehensive information at single-cell resolution and could help to explore yet unrecognized cellular activity both intracellularly and intercellularly. In addition, single-cell techniques could also help to identify biomarkers to determine TCP, tumor response to targeted therapy, as well as prognosis.^374^ Artificial intelligence could also be employed to capture the underlying regularities of such an unwieldly mass of data. It was expected that one day, with the help of big data and the corresponding interpreting capacity, complete cures could be achieved by personalized treatment strategies decided by molecules at single-cell level.

In the face of such capricious, unpredictable, and threatening disease, scientific research will be always on the way to distill the complexity of cancer into an increasingly logical science. The attempts to get a better understanding of TCP in targeted therapy hold great promise for future blockade against therapy evasion, which may promote realization of long-lasting curative effects and even complete cure in cancer patients. Ongoing clinical trials using conventional treatments combined with plasticity-regulating treatment also indicate new opportunities for achieving the goal of complete cancer cure.

## Supplementary information

Duplicate checking of papers

Editing Certificate1

Editing Certificate2
