# Supplementary material for: Tumor cell plasticity in targeted therapy-induced resistance: mechanisms and new strategies
Source: Signal Transduct Target Ther. 2023 Mar 11;8:113. doi: 10.1038/s41392-023-01383-x (PMC10008648; doi:10.1038/s41392-023-01383-x)
Supplement: Supplementary file 1 — Duplicate checking of papers [file 41392_2023_1383_MOESM1_ESM.pdf]

605824288976535552_11002_555FDC64649ABCCBF18DEC44CB0BAB40.txt

by

Submission date: 29-Jan-2023 09:20PM (UTC-0600)
Submission ID: 2002075857
File name: 605824288976535552_11002_555FDC64649ABCCBF18DEC44CB0BAB40.txt (75.92K)
Word count: 10827
Character count: 66789

Tumor cell plasticity in targeted therapy-induced resistance: mechanisms and new strategiesRunning Title: Drug-induced tumor cell plasticityAbstractDespite the success of targeted therapies in cancer treatment, therapy-induced resistance remains a major obstacle to a complete cure. Tumor cells evade treatments and relapse via phenotypic switching driven by intrinsic or induced cell plasticity. Several reversible mechanisms have been proposed to circumvent tumor cell plasticity, including epigenetic modifications, regulation of transcription factors, activation or suppression of key signaling pathways, as well as modification of the tumor environment. Epithelial-to-mesenchymal transition, tumor cell and cancer stem cell formation also serve as roads towards tumor cell plasticity. Corresponding treatment strategies have recently been developed that either target plasticity-related mechanisms or employ combination treatments. In this review, we delineate the formation of tumor cell plasticity and its manipulation of tumor evasion from targeted therapy. We discuss the non-genetic mechanisms of targeted drug-induced tumor cell plasticity in various types of tumors and provide insights into the contribution of tumor cell plasticity to acquired drug resistance. New therapeutic strategies such as inhibition or reversal of tumor cell plasticity are also presented. We also discuss the multitude of clinical trials that are ongoing worldwide with the intention of improving clinical outcomes. These advances provide a direction for developing novel therapeutic strategies and combination therapy regimens that target tumor cell plasticity.Introduction to tumor cell plasticity (TCP)Cellular plasticity is a fundamental feature of cells. During development, cells progressively differentiate into different hierarchies and obtain specific developmental commitments. Such processes are reversible and the phenotypic changes can be

diversely directed as needed. Genetic lineage tracing has demonstrated that functionally mature cells can undergo dedifferentiation (lineage reversion), transdifferentiation (cells converting into other distant lineages), or transdetermination (cells switching lineage commitment to a closely related cell type) upon injury or disease 1,2. As far back as 1956, Conrad Waddington stated that during development embryonic progenitor cells progressively differentiate into distinct cell types and are increasingly restricted within a specific lineage 3. The final specialized cells are then integrated into their respective tissues and conduct their specific invariable functions. However, later studies subverted this paradigm. Han Driesch isolated from a sea urchin embryo a single blastomere in an early multicellular state could develop into an entire larva 4. This finding suggested that partially developed cells are not fully determined, but retain the potency to produce all types of cells found in an entire organism. In fact, the states of cells are quite dynamic, especially when injury or disease occurs. An equilibrium between new cell generation and old cell loss is generally maintained in multicellular organisms. If perturbed by unexpected loss, stem cell replication and differentiation along with transformation of mature differentiated cells can restore equilibrium. For example, endothelial cells assume arterial, venous, or lymphatic identity in an early developmental stage. However, upon injury, endothelial cells can undergo lineage changes to become hematopoietic progenitor or cardiac valve cells 5-7. In liver homeostasis, modest proliferation of hepatocytes in all zones contributes to the homeostatic hepatocyte pool, rather than a rare type of liver progenitor cell (LPC) subpopulation 8,9. In a genetic mouse model which mimics human Alagille syndrome and contributes to biliary epithelium regeneration,

hepatocytes are able to transdifferentiate into biliary epithelial cells 10. Additionally, cellular plasticity can be “artificially” enhanced, as demonstrated by induced pluripotent stem cells (iPSCs), which were first generated in 2007 11, providing unprecedented opportunities for novel therapies.Cellular plasticity also contributes to pathological conditions. Similar to the cell plasticity observed during post-injury repair and regeneration, plasticity in tumor cells promotes survival against immune attack and anti-tumor treatments. A major obstacle preventing complete remission, this evasion to therapy occurs as cancer cells play “hide and seek” by constant phenotypic switching. Some regard this phenotypic switching of cancer cells to result from passive Darwinian selection and enrichment upon drug exposure 12,13, while others argue it is an active adaptation to internal or external stimuli (Lamarckian induction) 14. A coexisting model has also been formulated to illustrate that these two concepts complement and intertwine with each other (Figure 1) 14-16. The “hide and seek” behavior of cancer cells is known as TCP which is a process that re-activates various developmental programs to achieve phenotypic switching. TCP enables transformation of tumor cell identity that allows the cells to adapt to different microenvironments and circumvent the drug-targeted pathway. This subpopulation of tumor cells is characterized by its dormant and stem-like trait and termed drug-tolerant persisters (DTPs) 15. The “acquired inertia” of DTPs is regarded as a survival skill exposed by anti-tumor treatment that can fade away after a drug-free interval 17. Thus, with TCP, a reversible switching of phenotypes can be achieved between proliferative, metastatic tumor cells and dormant, drug-indifferent DTPs, thereby hindering the desired outcome of targeted therapies. By this universally plastic behavior, tumor cells transform in

different but functionally overlapping processes including epithelial-mesenchymal transition (EMT), transdifferentiation, and acquiring properties of cancer stem cells (CSCs) 14,18,19. Epithelial–mesenchymal transitionEMT is a process by which tumor cells lose their epithelial features and acquire mesenchymal characteristics. EMT is characterized by loss of apical-basal polarity and disruption of intercellular interactions, accompanied by the acquisition of front-rear polarity and remodeling of the cytoskeleton 20,21. On the other hand, mesenchymal-epithelial transition (MET) is the inverse process of EMT. EMT and MET are thought to be regulated mainly at the transcriptional level by the activity of EMT transcription factors (EMT-TFs), including Snail, Slug, Zeb1/2, Twist, and microRNAs (miRs). Signaling pathways such as transforming growth factor (TGF-β), wingless/integrated (WNT), NOTCH, and HIPPO are also involved 15,22. Two double-negative feedback loops that include Snail/miR-34 and Zeb/miR-200 help to manipulate the initiation, transition, and maintenance of EMT program 23,24. Genetic mutations may also play a role in EMT regulation. As indicated by Martin et al. 25, a combined loss of the tumor suppressor gene P53 and PTEN in clonal prostate epithelial cells can lead to transformation of multipotent progenitors and ultimately EMT. Yamasaki et al. 26 recently proposed that Kirsten rat sarcoma viral oncogene homolog (KRAS) mutation can also promote an EMT-like phenotypic change. Both EMT and MET have been proposed to play key roles in cancer progression 21. Apart from a complete EMT/MET transition, tumor cells can exhibit a “partial EMT” state, a hybrid of epithelial and mesenchymal phenotypes 27. In fact, tumor cells can exhibit a spectrum of EMT states characterized by varying proportions of epithelial and mesenchymal traits, both in circulation and at a secondary

site 28,29. Panchy et al. 30 recently confirmed this epithelial-mesenchymal spectrum using transcriptomic analysis. This hybrid epithelial-mesenchymal state enables tumor cells to self-adapt and adjust quickly to changing environments, thus representing an ideal mechanism of TCP 28. Previous studies indicate that dynamic changes between epithelial and mesenchymal phenotype in tumor cells with partial EMT are critically important to the metastatic potential and therapy resistance of tumor cells 31. The contributions made by EMT programs to metastasis were first proposed by Yang et al. 32, who described that inhibition of Twist, a classic EMT-TF, can significantly restrain highly metastatic mammary carcinoma cells from metastasizing to the lung. The impact of other EMT-TFs on metastasis was then extensively investigated in different types of cancers 33. For example, depletion of Zeb1 was found to markedly inhibit pancreatic ductal adenocarcinoma progression towards metastatic type 34. Zeb1 was also found to function as a key master regulator of multiple EMT-TFs and metastasis in breast cancer 33. The MET program, in contrast, presumably functions during metastasis when the migratory CSCs settle down and adapt to a new microenvironment. In the process of colonization, the migratory CSCs regain self-renewal capacity and spawn fast cycling epithelial progeny, thereby restoring epithelial characteristics 35. Such reversible EMT-MET programs seem to be a common feature shared by different cancer types during the seeding of a secondary tumor 35-37. Evidence also suggests that EMT can serve as a survival mechanism for cancer cells to escape immunosurveillance and resist therapy-induced death 38. Multiple studies have observed a strong correlation between the level of mesenchymal cell markers and therapeutic resistance 21,39-42. For example, Farmer et al. 42 demonstrated that an

upregulation of stromal gene expression predicted resistance to preoperative chemotherapy with 5-fluororacil, epirubicin, and cyclophosphamide in estrogen receptor (ER)-negative breast cancer. In ovarian cancer, inhibition of EMT can effectively eliminate cisplatin resistance 43. Byers et al. 40 revealed a correlation between a 76-gene mesenchymal signature and non-small-cell lung cancer (NSCLC) resistance to epidermal growth factor receptor-tyrosine kinase inhibitors (EGFR-TKIs) and phosphatidylinositol 3-kinase/protein kinase B (PI3K/Akt) inhibitors. By proteomic screening, Paulitschke et al. 41 also found that two EMT-related markers are effective in determining drug susceptibility in melanoma cells. Although the mechanisms underlying the therapeutic resistance conferred by EMT remain unclear, several hypotheses have been put forward, including reduced levels of proapoptotic proteins, increased drug efflux, and immunosuppressive tumor microenvironment (TME) 44-46. TransdifferentiationTransdifferentiation, also known as lineage plasticity or lineage switching, is a radical shift in cell identity that leads to cells acquiring new transcriptional or morphological characteristics 47,48. In the context of cancer, such lineage plasticity may serve as a promising therapeutic approach. For example, melanoma spheroid cells are able to transdifferentiate into benign cell lineages including melanocytes, adipocytes, osteocytes and chondrocytes 49. However, transdifferentiation in cancer cells can also be a double-edge sword. Transdifferentiation observed in basal cell carcinoma (BCC) actually contributes to BCC relapse following treatment discontinuation 50,51. When treated with vismodegib, some BCC cells transitioned from a phenotype featuring a transcriptional bulge-like signature to a mixed isthmus/interfollicular epidermis cell phenotype, thereby achieving

drug resistance 52. A discontinuation of treatment led to tumor cell reactivation and resumption of proliferation 52,53. Another distinctive form of transdifferentiation is characterized by a drug-induced neuroendocrine transdifferentiation (NET). Observed in prostate cancer (PC) and lung adenocarcinoma (LUAD), NET induces drug resistance, thereby causing treatment failure and relapse 14,54,55. Here, we consider PC and LUAD as two typical examples of NET for further discussion. Through NET, PC tumor cells gradually obtain the histomorphology of small cell prostate cancers (SCPC) that are “therapy indifferent” to androgen receptor signaling inhibitors (ARSi) 54,56. The switch from PC to SCPC is characterized by the presence of neuroendocrine markers (chromogranin A, synaptophysin, and others), and the absence of androgen receptor (AR) and serum prostate-specific antigen 57. Functionally, SCPC cells have an elevated metastatic potential, enhanced stemness, heightened therapeutic resistance, and a worse prognosis 18,58. Inactivation of TP53, RB1, PTEN, and amplification of MYCN have been reported to be involved at the molecular level of the cell identity shift 18,59. Mutations in these candidate genes provide a specific genomic background that enables epigenetic regulation towards tumor plasticity 48,60. Moreover, epigenetic events are also indispensable. NET of PC cells towards SCPC is associated with the well-established undifferentiated cell marker SRY-Box Transcription Factor 2 (SOX2), epigenetic regulator histone methyltransferase enhancer of zeste homolog 2 (EZH2), the catalytic subunit of the polycomb repressive complex 2 (PRC2), and RE1-silencing transcription factor (REST) 14,18,61-64. A recent study by Cyrta et al. 65 suggested that the switch/sucrose non-fermentable (SWI/SNF) chromatin remodeling complexes may also be a potential

regulator of SCPC lineage plasticity. Correspondingly, SCPC can be reverted to its original lineage by the re-establishment of TP53 and RB1 as well as re-exposure to androgens, or inhibition of SOX2, thus re-sensitized to ARSi treatment 61,62. This reversibility of lineage plasticity could be of critical importance as a future target in PC therapy. Like SCPC, LUAD undergoes NET through LUAD – small-cell lung cancer (SCLC) transition, which enables the tumor to escape from EGFR-TKI treatment and gain a higher level of malignancy 48,54,66. Although NET is also identified in non-EGFR-mutant NSCLC, EGFR mutant LUADs are more likely to switch lineage 67. Oser et al. 68 reported that 5%-15% of EGFR mutant LUAD patients with TKI resistance showed small cell histology in relapsed tumors, and became sensitive to SCLC chemotherapy 48. The mechanism of LUAD NET at the molecular level is similar to that of PC NET. LUADs with loss of TP53 and RB1 expression are more likely to transdifferentiate into SCLC tumors, which suggests that these two genes may be critical in promoting transdifferentiation towards a neuroendocrine identity 60,68-70. The epigenetic regulator EZH2 is also regarded as a potential therapeutic target to reverse NET 71,72. PC and LUAD are currently the most extensively studied cancers exhibiting NET; however, transdifferentiation of epithelial cells is more common. According to a recent study, many epithelial cancers including bladder, endometrial, and gastric cancer with neuroendocrine gene expression signatures tend to exhibit high-grade tumors and poor prognosis 66. This finding indicates that a latent neuroendocrine lineage plasticity may occur in multiple tissues. Cancer stem cellsAccumulating evidence suggests that the existence of CSCs within tumor tissues is responsible for tumor initiation and intertumoral heterogeneity. CSCs have the

potential to proliferate infinitely, self-renew, and differentiate 73,74. A recent study proposed that a CSC is a highly plastic state that results in fast adaptation of tumor cells to varying conditions in the TME 75. Previous studies revealed CSCs and adult stem cells share multiple signatures, including the surface markers CD133, CD44, CD24, CD26, CD166, and EPCAM, and intracellular proteins like ALDH1 76-78. Additionally, CSCs and stem cells also share similar developmental-related signaling pathways, such as WNT, NOTCH, and Hedgehog (Hh) 29,79.Like normal stem cells, CSCs propagate through asymmetric division, which produces one daughter cell that remains a CSC and one committed progenitor cell that differentiates into a non-CSC 80,81. Symmetric division can also occur simultaneously, with the production of either two CSCs (symmetric renewal) or two committed cells (symmetric commitment) 80,82. The transition between CSC and non-CSC states seems irreversible and dynamically balanced. Under certain circumstances, however, the balance can shift either direction. A shift towards renewal results in replenishment of the CSC pool and consequently produces a more aggressive, undifferentiated phenotype. A shift towards non-CSCs suggests tumor-formation 29. Equilibrium represents the plasticity of tumor cell fate and can be manipulated by various influencing factors. For example, a second mutation introduced in chronic-phase chronic myeloid leukemia (CML) has been found to trigger an imbalance that favors symmetric renewal and results in higher malignancy 83. EMT-TF Twist2 has been reported to induce stemness properties in lung CSCs by moving the balance towards symmetric division, while repressing asymmetric division 84. Other epigenetic regulations, including deoxyribonucleic acid (DNA) methylation, histone modification, and bivalent chromatin state, have been found to contribute to

CSC plasticity 78,85. These studies indicate that manipulation of CSC division may be promising for cancer control. CSCs and EMT share a high degree of consistency and may be a partially overlapping concept 86,87. As mentioned, the EMT program largely facilitates tumor cell dissemination. CSCs are also enriched in disseminated tumor cells and feature EMT expression signatures 88. Circulating tumor cells express a remarkably congruent transcriptomic profile with CSCs and have been found to overexpress both EMT and stem cell markers 89. CSCs lead to therapeutic resistance through various mechanisms. For example, CSCs can specifically inherit effective drug-discharging ATP-binding cassette (ABC) transporters from asymmetrical division, which enable tumors to sustain long-term expansion 90,91. CSCs can also facilitate efficient DNA damage 92-94. Another mechanism of therapeutic resistance in CSCs is the formation of a protective niche, an immunosuppressive microenvironment characterized by low infiltration of immune cells and downregulated signaling by the programmed cell death ligand 1(PD-L1) 79,95. Non-CSCs also help to maintain of a pool of CSCs by secreting supportive signals such as WNT and brain-derived neurotrophic factor (BDNF), as well as supportive factors such as interleukin (IL)-6 and IL-8 96-98. Mechanism of therapy-induced TCPVarious therapies have been developed to prolong survival and improve the quality of life of cancer patients, yet therapy-induced drug resistance remains a major challenge to achieving the desired clinical outcomes. Notably, up to 90% cancer-associated deaths are attributed to drug resistance 99. Acquired drug resistance can be induced by targeted treatments and is associated with TCP, the “hide-and-seek” behavior of cancer cells. The drug-resistant phenotype of cancer cells is not heritable, but is a transient and reversible process 16.

Therefore, a comprehensive understanding of the mechanism of such induced plasticity should greatly facilitate the development of novel therapies. Epigenetic alterations, transcription factors, key signaling pathways, and TME can each potentially contribute to the therapy-induced plasticity in multiple cancers.Epigenetic mechanismsTherapy-induced epigenetic plasticity largely contributes to the reprogramming of tumor cells and phenotypic switching towards therapy resistance. The epigenetic modifications can be either chemical (DNA methylation and histone modification) or structural (chromatin remodeling and inter/intrachromosomal interactions) 100,101 (Figure 2A).DNA methylation is widespread in the human genome and functions differently based on various methylated regions 99. The aberrant methylation status of gene promoters plays an instrumental role in acquired drug resistance in a variety of tumor types 102,103. The most common mechanisms of drug resistance include hypomethylation of gene promoters of drug efflux, hypermethylation of promoters of pro-apoptotic genes, and altered methylation levels of DNA-repair genes 99. Global loss of DNA methylation and histone acetylation has been observed in therapy-induced drug resistant tumor cells under extended treatment with the neoadjuvant aromatase inhibitor (letrozole). Such epigenetic alterations result in tumor cells escaping from dormancy and acquiring resistance after targeted therapy in breast cancer patients 104. In contrast, indifference or resistance to endocrine therapy in ER-positive breast cancer is due to hypermethylation of estrogen-responsive enhancers 105. Moreover, effective therapies targeting the AR in PC inadvertently promote NET. The lineage switching into neuroendocrine PC was associated with increased DNA methylation, turning the original PC into a lethal,

castration-resistant type. This process can be reversed by inhibition of DNA methyltransferase (DNMT) 106. Histone modification, represented by euchromatin histone lysine methyltransferase 2 (EHMT2), has been implicated in acquired drug resistance following targeted therapy. EHMT2 overexpression was observed in erlotinib resistance in NSCLC, leading to transcriptional activation of PTEN and repressed Akt signaling, whereas inhibition of EHMT2 reverses the acquired indifference to therapy and re-sensitizes NSCLC cells to EGFR-TKI treatment 107. Histone modifications, either via EHMT2 or histone deacetylase 3 (HDAC3), can result in acquired resistance against mitogen-activated extracellular signal-regulated kinase inhibitors (MEKi) treatment in pancreatic ductal adenocarcinoma (PDAC) 108. Structural epigenetic alterations, including changes in high-order structures of chromatin and interactions among distal regulatory elements, may also be involved in therapy-induced TCP. For example, targeted therapy using γ-secretase inhibitors in T cell acute lymphoblastic leukemias (T-ALL) can induce the expansion of a rare group of pre-existing persister cells. These persisters contain more compact chromatin and higher levels of repressive histone modifications compared to γ-secretase inhibitor-sensitive naïve cells 109. Similar to trametinib-resistant triple-negative breast cancer (TNBC), remodeling of enhancers and promoters was observed accompanied with adaptive transcriptomic responses 110.The role of transcription factors in controlling TCPA network of transcription factors also plays an important role in TCP against targeted therapy. EMT-TFs like Twist and Snail actively respond to targeted therapy during the EMT process and result in enhanced plasticity and mesenchymal switching 111,112. In addition to multiple EMT-TFs, several other transcription factors function in

controlling TCP. AR is known as a ligand-activated transcription factor which regulates gene expression in the prostate epithelium 113. AR targeted inhibitors, including bicalutamide and enzalutamide, have been developed for PC treatment 114,115. However, upon castration therapy, evading mechanisms are launched by AR amplification, point mutation, splicing variants, as well as substitution of AR functions by glucocorticoid receptors 116. Signal transducer and activator of transcription 3 (STAT3) is a transcription factor that has been studied extensively in multiple cancers. Previous studies have suggested that STAT3 activation is associated with trastuzumab as well as trastuzumab-emtansine resistance in human epidermal growth factor receptor 2 (HER2)-positive breast cancer 117,118. In triple negative (ER-/PR-/HER2-) breast cancer (TNBC) patients, a strong transcriptomic response was observed after treatment with trametinib for seven continuous days, with up to 22% of the transcriptome either significantly up- or downregulated 110. The super transcription factor cellular MYC underwent rapid degradation during the treatment process and functioned as a controller of genomic reorganization and transcriptional complex recruitment in the phenotypic change of tumors 110,119. In melanoma, an assay for transposase-accessible chromatin using sequencing (ATAC-seq) revealed an initial loss of transcription factor binding sites, followed by an increase in accessible sites, during BRAF inhibition (BRAFi) treatment. These results indicated that targeted therapy altered transcription factors 120. According to a recent study by Yang et al. 121, activating transcription factor 4 (ATF4) stress signaling led to rapid tumor evasion within a few days after the initiation of mitogen-activated protein kinase (MAPK) pathway inhibition, and was accompanied by elevated levels of microphthalmia-

associated transcription factor (MITF). This was followed by tumor cell dedifferentiation and a dormant phenotype marked with decrease in MITF expression. Additionally, the helix-loop-helix transcription factor inhibitor of DNA binding 3 (ID3) was upregulated after BRAFi therapy compared to that in the pretreatment state. ID3 acted as a transcriptional repressor of sex-determining region Y-box 10 (SOX10) and MITF, thereby promoting tumor cell transformation to a drug-resistant phenotype 122. Moreover, Sun et al. 123 also observed an increase in the percentage of SOX10low EGFRhigh melanoma cells after BRAFi and MEKi treatment. This subset of tumor cells displayed a slow-cycling phenotype in response to these targeted drugs, highlighting the potential of transcription factors to mediate plasticity in melanoma. The regulation patterns of the numerous known transcription factors are quite distinct in targeted therapy-induced tumor cell lines 120,124. This complexity indicates that tumors acquire resistance against targeted therapies in a patient- and drug-dependent manner. Hence, the transcription factors and their interaction modes remain to be explored. Key signaling pathways in controlling TCPNumerous signaling pathways are involved in embryonic development, and a re-activation of these signaling pathways is critical in tumor progression and therapy resistance. Interaction of these conserved signaling pathways (e.g., Hh, Wnt, and Notch pathways) and their crosstalk with other oncogenic pathways (e.g., Nuclear factor kappa B (NF-κB), PI3K/AKT/Mammalian target of rapamycin (mTOR), and MAPK pathways) contributes significantly to TCP by regulating the fluctuating expression of downstream effectors 125-128 (Figure 2B). Phenotypic switching driven by multiple signaling pathways is a huge challenge to successful tumor treatment.Alterations of the Hh (Hedgehog) signaling pathway occur

in one-third of cancers, and deregulation of the Hh signaling pathway plays an important role in therapeutic resistance 15,129-132. Aberrant activation of Hh signaling and subsequent upregulation of downstream effector glioma-associated transcription factor 2 (GLI2) was observed in bortezomib-resistant myeloma cells 133,134. Further studies demonstrated that Hh signaling is involved in CSC self-renewal in multiple myeloma, and blocking Hh signaling can lead to CSC differentiation 135. In TNBC, the Hh ligand produced by tumor cells can reprogram cancer-associated fibroblasts (CAFs) to promote a CSC-phenotype switching and the acquisition of chemo-resistant traits 136. Therefore, an effective Hh signaling pathway blockade is capable of lowering CSC proliferation, metastasis, and the EMT program, thereby controlling TCP. Wnt signaling pathway is also highly associated with TCP. Wnt signaling is complex and can function through either an autocrine or a paracrine mode, by canonical (Wnt/β-catenin pathway) or non-canonical (the planar cell polarity pathway) pathways. Wnt signaling contributes to EMT regulation and TCP 137. In most cases, the Wnt/β-catenin signaling pathway is constitutively activated, which increases the expression of Snail, a regulator of EMT. Inhibition of the Wnt/β-catenin pathway inhibits EMT and tumor metastasis in a colorectal cancer model 138. Slug is another EMT modulator that is considered to be a downstream protein in Wnt/β-catenin-mediated tumor invasion. Moon et al. reported that concurrent expression of β-catenin and Slug predicts lymph node metastasis and survival rate in patients with head and neck squamous cell carcinoma 139. Overexpression of Slug can induce cancer cell stemness and enhance cell invasion and metastasis. Sun et al. reported that Slug overexpression is associated with the invasiveness of liver cancer both in vitro and in

vivo 140. In contrast, overexpression of Snail did not have a similar effect in liver cancer cells. The Wnt pathway has been linked to increased expression of another EMT transcription factor, Twist, in mammary epithelial cells 141,142. Accumulating data show that TWIST not only induces EMT, but also promotes the formation of CSCs and enhances tumorigenesis 143. Therefore, inhibiting these EMT mediators may represent the strategy for inhibiting tumor metastasis and recurrence. Additionally, activation of the Wnt signaling pathway reportedly controls the stemness of CSCs in different types of cancers including hepatocellular carcinoma (HCC), and colon, lung, and other cancers 144-148. According to clinical practice, targeted treatment using MEKi (selumetinib, trametinib, and PD318088) inadvertently increased Wnt activity and led to enhanced stemness and tumor relapse in colorectal cancer 149. Similarly, valproic acid, which was originally regarded as a histone deacetylase (HDAC) inhibitor in breast cancer treatment, can activate Wnt signaling pathway and promote the pool of mammary CSCs, leading to tumor progression 150. Hence, attention should be paid to unexpected phenotypic switching regulated by Wnt signaling pathway in the processes of targeted therapy. The Notch signaling pathway also enriches CSCs and regulates the EMT program in multiple cancers, including breast and lung cancer, pancreatic ductal adenocarcinoma, and malignant mesothelioma 151-155. A previous finding showed a change in EMT status in lung adenocarcinoma upon treatment with gefitinib 156. Subsequent studies further confirmed that the EMT process resulted from the activation of Notch-1 signaling pathway and led to gefitinib-acquired resistance 157. Notch signaling is one of the master regulators of neuroendocrine transdifferentiation in NSCLC, which enables NSCLC to transdifferentiate into SCLC

and acquire resistance to EGFR-TKIs after initial targeted treatment 154. By extension, the Notch pathway has been discovered to play a crucial role in the EMT process in prostate cancer cells, promote a transformation towards basal stem-like properties, and lead to a castration-resistant phenotype 158,159. Components of the Notch signaling pathway are expressed at significant levels in tumor invasive regions, which suggests its crucial role in EMT regulation. A recent paper by Natsuizaka et al. reported that Notch1 signaling and EMT work closely to promote squamous cell carcinoma tumor initiation. Moreover, another important protein, TGF-β, guides Notch1 to drive EMT 160. A study by Zeng et al. showed that inhibition of Notch1 reverses the EMT process and overcomes cisplatin resistance in breast cancer cells 161. The data also suggested that high expression of Notch1 predicts a poor prognosis in TNBC patients. Saad et al. demonstrated that the Notch-mediated activation of EMT is associated with the regulation of EMT transcription factors 162. The two transcription factors, Snail and Slug, are involved in the inhibition of E-cadherin and β-catenin mediated by the Notch pathway. Inhibition of Notch activation limits the increase of MMP-2 and -9 expression, while it decreases Snail expression. The study provided direct evidence that Notch signaling induces EMT via the Snail pathway. Surprisingly, Notch signaling can synergistically work with other pathways to facilitate the EMT process. The crosstalk between these pathways is mediated by suppressor of mothers against decapentaplegic (SMADs) proteins that associate with other transcription factors for regulating the expression of genes required for the transition to mesenchymal characteristics. A single-cell sequencing study by Deshmukh et al. revealed both sequential and parallel activation of EMT signaling pathways. The Notch

pathway also functions as a crucial regulator of TGF-β–induced EMT 163. The role of TME in controlling TCPTME is a key player in TCP induced by targeted treatment. The characteristics of TME, including immune and stromal cells, cytokines, exosomes, hypoxia, pH change, nutritional deficiency, and angiogenesis, are key regulators of TCP 164-167. CAFs are recognized as major contributors in the crosstalk between tumor cells and adjacent stromal cells 168. By producing a variety of bioactive molecules such as growth factors and cytokines, CAFs exert a profound impact on TME and influence TCP 96,169,170, and have long been described as crucial players in the EMT program. CAFs also participate in CSC pool expansion 171-173. The effect of CAFs in manipulating therapy resistance was reported by Apicella et al. 174. In this study, a prolonged treatment with TKIs generated resistance to erlotinib in vivo, but not in vitro. This intriguing phenomenon was explained by the existence of CAFs. Erlotinib treatment in vivo induces a metabolic rewiring towards aerobic glycolysis and increases lactate production; the secreted lactate triggers the overproduction of hepatocyte growth factor (HGF) in CAFs. Engulfed lactate and the subsequently increased HGF then activate the MET signaling pathway in cancer cells and cause resistance to erlotinib 175-177. Moreover, hypoxia-induced glycolysis represses natural killer (NK) cells via lactate accumulation and reduces cytotoxicity by decreased level of NKG2D, CD16, perforin, and granzyme B, thereby forming an immunosuppressive TME. Such hypoxia-related TME conditions were reported to play a role in resistance to therapeutic monoclonal antibodies 178,179.Cytokines secreted by immune and stromal cells also provide clues for acquired TCP induced by targeted therapy 180-182. According to Li et al. 183, IL-6 in EFGR-mutant NSCLC patients was

significantly increased upon osimertinib therapy. This associated Upregulation of laminin α5, which is among the most widely distributed laminins and is a major component of extracellular matrix (ECM), also occurred. By upregulation of laminin α5, IL-6 remodeled ECM and activated a downstream target focal adhesion kinase (FAK), thus contributing to acquired resistance against Osimertinib 183-185. FAK activation was confirmed to be dispensable for IL-6 production in maintenance of mesenchymal stem cells and can lead to acquired resistance of gefitinib as well 186,187.

Contribution of TCP to targeted therapy resistanceTargeted therapy is one of the most successful treatments that strike at the heart of a wide range of cancers. Nonetheless, the emergence of drug resistance significantly impairs the efficacy of targeted drugs and contributes to cancer mortality. Drug target mutations and activation of bypass pathways are known to confer resistance in cancer cells to targeted inhibitors. With an increasing understanding of TCP, it is now recognized that TCP is an important factor in mediating drug resistance. In this section, we focus on the role of TCP in shaping tumor cell characteristics and mediating targeted therapy resistance (Figure 4).Epithelial–mesenchymal transition related factors in mediating drug resistanceAs described above, EMT plays a critical role in mediating TCP and resistance to targeted therapy. The principal mechanisms of EMT-related drug resistance can be attributed to slow cell proliferation, increased drug efflux, and escape from apoptosis. Lung cancer is by far the most well-documented subtype of cancer that links EMT and targeted drug resistance. Namba et al. demonstrated that the upregulation of EMT-related gene AXL contributes to acquired resistance to osimertinib 188. Other EMT-related transcription factors, such as Snail, Slug, and ZEB1/2, are also reported to cause TKI resistance by

regulation of EMT-related signaling pathways 189-191. Therefore, a comprehensive understanding of the EMT process is critical to combat targeted therapy resistance. In this subsection, we delineate the current research on signaling pathways and related factors that modulate EMT associated with drug resistance.Signaling pathways including Notch, Wnt/β-catenin, and TGF-β play crucial roles in activating EMT-related transcription factors 192. Evidence supports that EMT transcription factors are involved in targeted therapy resistance, especially towards EGFR inhibitors. Consequently, signaling pathways that regulate the EMT process are important in mediating drug resistance. Chang et al. reported that Slug contributes to gefitinib resistance in NSCLC cells through downregulation of Bim and inhibition of caspase-9 activity 193. Among EMT inducers, TGF-β is significant because of its potency in inducing EMT and its roles in cancer-associated EMT 194. TGF-β-induced EMT is essential in cellular activities such as glycolysis and lipid/choline metabolism reprogramming 195. In response to TGF-β, cancer cells undergo complete or partial EMT, where epithelial and mesenchymal markers co-exist on cancer cells 37. The partial EMT status is characterized by an aggressive phenotype that renders cancer cells with high stemness and cell plasticity 196. Upon stimulation of TGF-β receptors, TGF-β signaling is divided into SMAD-dependent and -independent pathways. Both TGF-β/SMAD and non-SMAD pathways play a significant role in mediating EMT and promoting cell plasticity 197. Additionally, signaling pathways such as PI3K/AKT, mTOR, and MAPK are reported to play essential roles in TGF-β-induced EMT 198,199. Surprisingly, TGF-β-induced EMT can be reversed. A study conducted by Katsuno et al. found that upon TGF-β removal, mesenchymal cells revert to an

epithelial cell phenotype 200. Moreover, prolonged TGF-β exposure promotes a stable EMT process in cancer cells, which is accompanied by increased tumor stemness and cancer drug resistance. The authors also identified that mTOR inhibitors can be a promising agent to target CSCs by antagonizing the TGF-β-induced EMT process.MicroRNA (miRNAs) are small non-coding RNAs that play a pivotal role in the regulation of both EMT that promotes tumor metastasis and MET that facilitates metastatic colonization 201. EMT-related miRNAs are documented to impact cancer cell stemness and drug resistance. Recent evidence showed that miRNAs may affect the EMT process by targeting related transcription factors including Snail, Slug, Twist, and ZEB1/2 202. The expression level of the Snail transcription factor may be controlled by multiple metastasis-related miRNAs, including miR-153, miR-203, miR-204, and miR-34c 203. The miR-30 family are well-documented mediators of EMT transcription factor Snail in different cancer subtypes, including lung and breast cancer 204. Liu et al. showed that miR-1 and miR-200 can inhibit EMT and mesenchymal differentiation via Slug-dependent mechanisms 205. Other newly identified EMT-related miRNAs include miR-27b-3p 206, which promotes the generation of circulating tumor cells, and miRNA-490-3p, which promotes the metastasis of invasive ductal carcinoma 207. Nonetheless, recent studies have reported that some miRNAs, such as miRNA-128 MiRNA-155-5p 208,209, are able to hinder the EMT process through inhibition of diverse EMT-related signaling pathways. With the increased understanding of miRNAs in EMT and TCP, different strategies focused on hindering miRNA function or miRNA delivery into tumor cells can be utilized to overcome cancer drug resistance.Tumor heterogeneityTumor heterogeneity is defined as the existence

of a subpopulation of cancer cells with difference genotypes and phenotypes that exhibit various characteristics within a primary tumor. Heterogeneity may lead to divergent sensitivity to treatment and facilitate the development of drug resistance 210. The most recent tumor heterogeneity model is known as the CSC plasticity model 211. CSC is a subpopulation of cancer cells with self-renewal capacity to switch between stem and differentiated states. This dynamic transition can be attributed to gene mutations, epigenetic modification, as well as changes in TME. It should be noted that the EMT signaling pathway plays a central role in mediating CSC transition, which promotes cancer metastasis, recurrence, and drug resistance. Morel et al. reported that the induction of EMT can enhance human breast epithelial cell stemness and tumorigenic characteristics 212, which has also been confirmed in colorectal, pancreatic, and liver cancers 213-215. Quintana et al. demonstrated that most of the phenotypic heterogeneity in melanoma can be explained by the phenotypic plasticity model 216. Although they did not identify a marker that can significantly distinguish tumorigenic from non-tumorigenic cells, CD271+ cells were shown to have little tumorigenic activity. However, the results were inconsistent in different studies 217, suggesting that the role of cell plasticity in tumor heterogeneity may vary in cancer subtypes. Another study carried out by Charles et al. showed that nitric oxide can enhance the tumorigenic capacity of platelet-derived growth factor (PDGF)-driven glioma cells in vivo; therefore, it reversibly promotes their ability to form tumors 218. Nakano et al. reported the dynamic equilibrium between CSCs and non-CSCs in the development of colorectal cancer. Such equilibrium is mainly controlled by EMT-related TGF-β signaling pathway 219. One study demonstrated that, in colon cancer

cells, the ablation of LGR5+ CSCs allowed the cancer cells to dedifferentiate into CSCs, which highlights the distinct CSC dependency for primary and metastatic tumor growth 220.Multiple studies showed that a high level of tumor heterogeneity is associated with poor response to targeted drugs including osimertinib, rociletinib, and gefitinib 221-223. Chabon et al. demonstrated that multi-drug resistance was observed in 46% patients after targeted therapies, suggesting a frequent intra-tumor heterogeneity 222. TCP, i.e., phenotype switching between different signaling pathways, can potentially lead to heterogeneity under therapeutic selective pressure. As mentioned above, cell plasticity is essential in multiple crucial biological 44 processes, including embryonic development, wound healing, and tissue regeneration. In tumors, the reactivation of these mechanisms allows tumor cells to switch between non-CSC and CSC-like phenotypes, leading to tumor progression, metastases, and drug resistance 224. Easwaran et al. performed a genome-wide analysis and found that silencing abnormal DNA methylation of promoters can decrease changes of plasticity of cell population and help in locking cell clones with abnormal retention of self-renewal capacity 225. Therefore, an effective approach to disrupt TCP may represent a promising strategy to tackle tumor heterogeneity and overcome drug resistance.Interplay between TCP and TMEA tumor is not only a group of cancer cells, but also 16 a complex interaction between cancer cells and their surrounding environment, known as the tumor microenvironment (TME), which has attracted substantial research and clinical interest in recent years 226. TME consists of tumor, immune (macrophages and lymphocytes), and stromal (stromal fibroblasts and endothelial cells) cells, blood vessels, and extracellular matrix (ECM) 227.

Accumulating data show that cancer cells are able to control the function of cellular and non-cellular components through complex signaling pathways 228. The cell-cell and cell-ECM interactions force non-malignant cells to acquire new phenotypes that facilitate the formation, progression, and metastasis of tumor cells. In addition, theinteractions of cancer cells with their TME are crucial to stimulate tumor heterogeneity, clonal evolution, and mediate drug resistance 229. TCP allows tumor cells to switch phenotypes and survive treatment through adaptive strategies, and the TME is involved in mediating TCP 230. For example, both cancer-associated fibroblasts (CAFs) and hypoxia contribute to oxidative stress, which is an important mediator of multiple adaptive strategies. Hypoxia is a hallmark of the TME and strongly linked to malignant progression, poor clinical response, and therapy resistance 231. Recent studies have shown that hypoxia may induce phenotypic switching, leading to drug resistance. Terry et al. reported that hypoxic stress induces phenotypic diversity of EMT in lung cancer 232, and leads to changes in EMT transcription factors such as SNAI1, SNAI2, and ZEB2, which ultimately result in the resistance to cell-mediated cytotoxicity. Paolicchi et al. suggested that tumor hypoxia is a major contributor to drug resistance and a predisposing factor for increased CSC formation and metastases 233. Therefore, one promising cancer treatment strategy is to block the proteins related to hypoxia-induced factor (HIF)-1α, EMT, and mitochondria functions, which in turn inhibits the response to hypoxia. In addition, cytokines such as interferon-gamma and tumor necrosis factor-alpha are believed to enhance stromal cell plasticity and contribute to TME 234. The interaction between TME and TCP can significantly contribute to the genetic heterogeneity 235. Reynolds et al. demonstrated that the

number of genetic mutations arising in cells within tumors is much higher than that in the laboratory culture 236. Their results provide strong evidence that TME promotes genetic instability during tumor progression. On the other hand, tumor heterogeneity can lead to TME reprogramming and thus give rise to cancer drug resistance 237. Taken together, the interplay between TCP and TME serves 7 an important role in mediating tumor progression, heterogeneity, and drug resistance. New strategies for targeting TCPPreventing initiation of TCPStrategies that target TCP include intermittent treatments, combination therapies, and targeting the key nodes of phenotypic switching. A study showed that intermittent treatments 3 and off dosing schedules may double the time of melanoma response to vemurafenib 74. However, intermittent treatment strategies are usually difficult to design as drug resistance may be complex and come from multiple mechanisms other than TCP 238,239. Combination therapies have also been applied to target parallel activation of key signaling pathways such as Hippo/yes-associated protein (YAP), PI3K, and EGFR 240,241, resulting 51 in a significant decrease in the number of residual tumor cells. However, combination therapies usually require more optimized drug administration protocols 3 to maximize responses and limit toxicity due to their complexity. Other strategies to prevent TCP have focused on inhibiting the key nodes of phenotypic switching genes. 3 Histone demethylases, KDM5A/B and KDM6A, were known to play significant roles in DTP survival 242-244. KDM inhibitors have been developed; however, they are still under assessment for their specificity and efficacy 245-248. DTPs undergo transcriptional adaption through changing their global chromatin landscape. Another similar strategy that targets 3 the bromodomain and extra terminal

domain (BET) BRD4 gene overcame trametinib resistance in triple-negative breast cancer, and was efficient both in vitro and in vivo 110. In basal cell carcinoma, the assessment of inhibition or activation of Wnt and Notch signaling pathways are under performing in clinical trials to treat pathway-dependent malignancies 249. Notch-activated tumor cells tend to undergo apoptosis upon vismodegib treatment. Inhibitions of Retinoid X receptor (RXR) pathways using HX531 together with MAPK inhibitors can significantly delay development of resistance to MAPK inhibitors, compared to treatment with MAPK inhibitors alone 238. Discovery of more drug targets that prevent tumor plasticity will enable the design of treatment strategies. Targeting the fate of new cellsIn addition to preventing the formation of cell plasticity, targeting the emerging drug-resistant new cell identity holds great potential for overcoming tumor plasticity (Figure 4). Transformation from the NSCLC to SCLC usually causes strong drug resistance due to cell plasticity 250. SCLC cells often lose EGFR expression; therefore, they are resistant to EGFR inhibitors, in contrast to the nontransformed NSCLC (specifically LUAD). Transformed SCLCs were more responsive to platinum-etoposide, similar to de novo SCLC 251. However, compared to de novo SCLC, transformed SCLC showed a higher response rate to taxanes yet were resistant to checkpoint inhibitor therapy, which was similar to EGFR-mutant LUAD 252. Recently, molecular studies showed that the growth of some mesenchymal cancer cells depends on the expression of phospholipid glutathione peroxidase 4 (GPX4), and inhibition of GPX4 can profoundly decrease DTP, thus preventing tumor relapse 253. However, current GPX4 inhibitors are chloroacetamide-containing compounds (such as RLS3), which have been identified to possess only poor pharmacokinetic properties, such as

low stability, promiscuity, and bioavailability. The GPX-knockout mouse was lethal, indicating that choosing the therapeutic window that targets this protein is challenging 254-256. Thus, finding drugs that target the new cell identity is necessary for designing therapeutic strategies.   Reversing TCP TCP can be driven by an inflamed and suppressive TME. Drugs and therapies have been developed against intracellular signaling kinases and extracellular signaling molecules in TME 257,258. Additionally, since epigenetic changes contribute to TCP, targeting those processes could potentially re-sensitize tumor cells to drug treatments. IL-8IL-8, a pro-inflammatory cytokine, is known to promote tumor cell remodeling and is involved in regulation of tumor cell stemness, EMT, and therapy resistance in multiple types of tumors. 259,260. IL-8 production promotes tumor cells to transform to a mesenchymal phenotype and increase tumor cell migration, leading to metastases 261. C-X-C chemokine receptor 1 (CXCR1) antagonist therapies have been found to reverse the IL-8 related events 262. Resistance to PI3K/Akt/mTOR pathway inhibition in hepatocellular carcinoma has been associated with both liver cancer stem cells and a high level of IL-8 expression. Rapamycin, the mTOR inhibitor, was able to decrease the IL-8 expression, thus decrease the resistance to sorafenib 263. A neutralizing antibody, HuMAX-IL8, was used to deplete IL-8 secretion and treat human TNBC 264. Another study showed that neutralizing IL-8 can alleviate resistance to erlotinib in NSCLC, thereby enhancing sensitivity in chemotherapy resistant cells 265. Inhibition of CXCR2 with SB225002 also alleviated the IL-8 related resistance in sorafenib-resistant ovarian tumor cells 266. TGF-βReversing EMT by blockage of TGF-β signaling is acknowledged as a promising way to reverse TCP, as TGF-β has been widely

recognized as the major inducer of EMT 267. A recent single-cell transcriptome-based study showed that blocking TGF-β1 could restore stromal plasticity in tumors by affecting a subset of interferon-licensed fibroblasts 268. However, the pleiotropic functions of TGF-β1 in cancer render therapeutic inhibition of this pathway challenging 269. TGF-β1 inhibition in various combination therapies that target the drug-induced plasticity is under evaluation 270. Galunisertib (LY2157299 monohydrate), a small molecule inhibitor that inhibits TGFβRI ALK5 kinase, has been used in numerous preclinical trials 271. Forskolin and cholera toxin have been identified through a drug screen for E-cadherin promoter activation 272. JMF3086, a dual inhibitor that targets both HDAC and 3-hydroxy-3-methylglutaryl coenzyme A reductase, can restore the sensitivity of NSCLC to EGFR-TKIs (34). A preclinical study showed that galunisertib has the potential to regulate pancreatic cancer cell growth through prevention of TGF-β mediated E-cadherin downregulation 273. Galunisertib also demonstrated anti-tumor efficacy in patients with pancreatic cancer as well as in a subset of patients with advanced HCC 274. A recent study also demonstrated that inhibition of TGF-β and leukocyte associated immunoglobin like receptor-1 (LAIR-1) can remodel TME, thereby enabling programmed-death ligand 1 (PD-L1)-mediated tumor eradication 275. Dual-blockade of CD73-TGFβ promotes a multifaceted inflammatory environment in TNBC, by diminishing myeloid-derived suppressor cells and M2-macrophages but increasing cytotoxic T cells and activated dendritic cells 276. These studies and novel drugs have provided strong evidence that blocking TGF-β can reverse TCP, thereby sensitizing tumor cells to targeted or immune mediated cell death. EZH2EZH2, the catalytic subunit of polycomb repressive complex 2 (PRC2), is the central player in the

process of epigenetic gene silencing. EZH2 promotes tumor cell survival, proliferation, EMT, and invasion, as well as drug resistance 277. Reversible transition between EMT and MET are key aspects of TCP. Interestingly, EZH2 was recently found to support TCP through MET facilitation and tumor colonization 278. Overexpression of EZH2 was associated with the conversion of prostate adenocarcinoma to neuroendocrine prostate cancer (NEPC), which is a more aggressive variant 279,280. Inhibition of EZH2 can reverse the cell lineage switch, leading to a decrease in neuroendocrine related key factors and restoring the sensitivity of NEPC cells to enzalutamide. Some EZH2 inhibitors (for example, CPI-1205 and Tazemetostat) have entered phase I and II clinical trials for lymphomas and solid tumors 281,282. Other targets and pathwaysOther targets and pathways with potential ability to reverse TCP are emerging. Ferroptosis was identified as a new type of programmed cell death that can be harnessed to target drug resistance and EMT. Gagliardi et al. identified that EMT-associated gene expression reprogramming process that determines ferroptotic susceptibility cannot be applied to metastatic-derived cells, but SCL7A11 was a valuable marker to predict the susceptibility 283. Increased sensitivity to ferroptosis through crosstalk of BACH1 target genes can also impose programmed vulnerability upon cancer cells 284. Other than ferroptosis, HDAC inhibition was recently found to reverse EBV-induced dedifferentiation in nasopharyngeal carcinoma 285. The Yap-Sox9 axis was recently reported to determine hepatocyte plasticity in hepatocarcinogenesis and could be a promising target 286. Some other SOX factors have also been considered as promising candidates through proteolysis-targeting chimeric molecules or thalidomide analogues 287,288. An anti-CLDN6 antibody-drug

conjugate has been reported to target tumor lineage plasticity 289. Another recent study showed that blocking the MNK1/2-eIF4E axis could inhibit melanoma phenotypic switching, thereby, sensitizing melanoma to anti–PD-1 immunotherapy 290. These studies suggest that reversing TCP through other targets could be a propitious future direction. New therapies for overcoming TCP-induced drug resistanceTCP enables tumor cells to evade traditional anti-tumor therapies. Therefore, agents that block these escape routes can deprive the residual tumor cells of their hiding place and ensure positive treatment results (Figure 5). With immune checkpoint blockade (ICB)An immunosuppressive TME is formed upon drug exposure as a consequence of the interaction between non-tumor and tumor cells. It is worth mentioning that the expression of immune checkpoint proteins including programmed cell death 1 receptor (PD-1) and (PD-L1), cytotoxic T lymphocyte associated protein 4 (CTLA-4), T cell immunoglobulin 3 (TIM-3), lymphocyte activation gene 3 (LAG-3), and T cell immunoreceptor with Ig and ITIM domains (TIGIT) contribute significantly to immunosuppression and serve as a bridge between EMT programs and immunosuppression 291-295. A positive correlation between EMT and immune checkpoint proteins was identified in multiple cancer types, including lung, breast, colorectum, oral, and esophageal cancers 293,296-301, and a higher expression of these checkpoint molecules indicates worse prognosis in a broad range of cancers 302-304. The functional relationship among immune checkpoint proteins and therapy evasion suggests that a combination treatment with immune checkpoint blockade (ICB) could be promising in developing a new approach against TCP. Combining targeted therapy with ICB seems feasible according to current practice. Traditional

EGFR-TKI was found to interfere with TME, thus laying a theoretical foundation for ICB application in EGFR-TKI-resistant NSCLC, especially in those with upregulated PD-L1 expression 305-307. Moreover, recent studies revealed that dynamic changes occur in TME in response to EGFR-TKIs and suggested that a combination treatment with immune-mediated anticancer approaches could be beneficial 306,308-311. EGFR-TKI-ICB combination treatment provided better clinical benefit for NSCLC patients with increased overall response rate (ORR) (41.7% vs 14.3%) and median progression free survival (PFS) (19.5 months vs 1.4 months) in comparison to monotherapy of either pembrolizumab or gefitinib alone 310. Patients with short EGFR-TKI-PFS may even benefit more from a combination therapy as a second-line treatment 312. A similar combination strategy was applied in multiple cancer types as a so-called basket trial design 313. A combination of ICB with antiangiogenic molecular targeted therapy is an attractive approach for treating HCC. According to the IMbrave150 trial, atezolizumab in combination with bevacizumab showed greater benefits compared with sorafenib in advanced HCC patients and suggested that this combination strategy could be used as the first-line standard of care 314-317. Additionally, a combination of ICB with other targeted therapeutic approaches including ATR kinase inhibition and androgen receptor blockade resulted in synergistic therapeutic response in prostate cancer 318-320. Combining chemotherapy with ICB can be used to extend a synergistic therapeutic effect 321,322. A pooled analysis of three randomized clinical trials indicated that among patients with PD-L1-negative advanced / metastatic NSCLC, efficacy is better with pembrolizumab plus chemotherapy compared with chemotherapy alone 323. Similar results were obtained in PD-L1-positive NSCLC

patients. As indicated by Zhang et al. 324, the combination regimen of toripalimab and pemetrexed/carboplatin was superior to chemotherapy alone. Moreover, an IMpower150 trial showed that a combination of atezolizumab, bevacizumab, carboplatin, and paclitaxel (ABCP) led to significant improvement in median overall survival (OS) in chemotherapy-naïve patients with NSCLC 325. Anti-CTLA-4 ipilimumab was also tested in the treatment for NSCLC in combination with nivolumab and platinum doublet chemotherapy and exhibited clinical value as a first-line treatment 326. Another phase Ⅲ clinical trial compared the efficacy of chemotherapy with nivolumab and chemotherapy alone in treatment for advanced gastric, gastroesophageal junction (GEJ), and esophageal adenocarcinoma, and found that the combination strategy had a benefit of significantly superior OS and PFS 326. Janjigian et al. 326 proposed that this combination treatment represented a new first-line therapy for patients with upper gastrointestinal tumors. A recent phase Ⅲ study (KEYNOTE-811) also confirmed that adding pembrolizumab to standard first-line treatment (trastuzumab plus chemotherapy) in advanced gastric or GEJ adenocarcinoma could markedly improve prognosis 327. Though the basket trial design has greatly improved the clinical outcome of multiple cancer types, our understanding of the underlying mechanisms of the combination regimens remains limited. For example, nab-paclitaxel combined with atezolizumab are able to effectively prolong PFS in patients with metastatic TNBC as shown by the IMPASSION130 trial; however, the combination of paclitaxel with atezoliaumb failed in the IMPASSION131 trial 328,329. Single-cell analyses revealed that baseline CXCL13+ T cell largely determined the clinical response in TNBC treatment 330. Further studies are

warranted to explore the exact mechanisms of different combination strategies to be able to pin-point patients with higher therapeutic potential. Furthermore, novel combination methods are emerging. For example, a bioengineered platelet was designed that combines an inner-loaded doxorubicin and outer-anchored anti-PDL1-crosslinked nanogels. This composite design was reported to have the potential to reduce the recurrence and metastasis rate of postsurgical tumors 331.Despite some optimistic reports, the safety profile of combination therapies with ICB remains poorly understood and controversial. In NSCLC treatment, an increased incidence of interstitial lung disease-like events was observed in the combination treatment of osimertinib and durvalumab in the CAURAL (38%) and TATTON (22%) trial 332-337. Severe hepatotoxicity was observed in another clinical trial (CheckMate370) investigating the combined effect of nivolumab and crizotininb 310. Similar hepatic and immune-related adverse events were also observed in combined treatment with ICBs in melanoma 338-340.These described studies imply that a combination with ICB can improve therapeutic outcomes for therapy-resistant patients compared with either therapy alone. The clinical feasibility of the combined strategy warrants further exploration and the safety profile should be carefully documented. Table 1 provides a list of some relevant clinical trials evaluating the combination therapy with ICB mentioned above.With EMT-modulating agentsBecause EMT plays an essential role in TCP and drug resistance, EMT-targeting compounds hold great potential as novel drugs in reversing drug resistance. However, investigation in this field is limited. EMT-TFs play an essential role in TCP, and drugs directly targeting these transcription factors might have the potential to halt or reverse the plasticity and become a novel

therapeutic approach to repress drug resistance. However, due to the redundancy of overlapping pathways and multitude of factors, it is hard to target and control TCP effectively. Direct targeting of these EMT-TFs is very challenging and there is no yet known EMT-TF-targeting molecule 19. Nevertheless, some related molecules involved in redundant EMT pathways have been investigated for clinical use. As a downstream target of YAP and EMT-TF Snail2, anexelekto (AXL) represents an ideal focus of EMT control 341,342. AXL-targeting inhibitors (SGI-7079 / Foretinib [XL-880] / Bemcentinib [BGB324] / 20G7-D9 / MYD1-72) have been reported to have an outstanding synergistic effect with erlotinib in NSCLC 40,343,344 and advanced pancreatic and ovarian cancers 341, BRAF inhibitors in melanoma 345, and temozolomide with radiation in glioblastoma 346. Multi-target AXL inhibitors like SGI-7079 and sunitinib have also shown prominent clinical value in reversing therapeutic resistance 40,347,348. Moreover, secreted clusterin, an EMT-related protein which was shown to be significantly upregulated in EMT progression and HCC migration, is a candidate target for HCC therapy 349-351. An antibody against secreted clusterin, termed as AB-16B5, was assessed in a phase Ⅱ clinical trial in combination with docetaxel for the treatment of metastatic NSCLC (Table 2).Inhibition of EMT-related pathways is a theoretically feasible approach to control TCP. TGF-β, a widely accepted key promoter of EMT and sustainer of mesenchymal and CSC states, contributes to the induction of immunosuppressive TME via hijacking Tregs and NK cells 44,45,352. A small molecule inhibitor of TGF-β receptor 1 named galunisertib (LY2157299 monohydrate) is attracting attention. One preclinical evaluation of galunisertib showed an inhibition of TGF-β-mediated downregulation of E-cadherin in mouse pancreatic cancer cell line,

indicating an anti-EMT effect 353. A recent study by Song et al. 354 demonstrated a synergistic effect in castration resistant PC using enzalutamide combined with galunisertib. The combinatorial galunisertib treatment successfully sensitized PC cells to traditional androgen deprivation therapy (ADT) and provided a novel therapeutic strategy for control of lineage transdifferentiation. Other studies tested the efficacy of galunisertib in HCC and indicated a prolonged OS using a combination of galunisertib and sorafenib 355. Melisi et al. 356 stated in a phase Ib/II clinical trial that a galunisertib-gemcitabine combination therapy could significantly improve OS in patients with unresectable pancreatic cancer compared with monotherapy with gemcitabine. The same result was found in myelodysplastic syndromes (MDS); galunsertib treatment was associated with hematologic improvement in patients with signs of blockage of early stem cell differentiation. However, some other clinical trials failed to prove the efficacy of galunisertib in HCC, metastatic pancreatic cancer, or glioma 357-359. Though such novel drugs can manipulate TCP and potentially re-sensitize tumor cells to immune-mediated cell death, further clinical trials are needed to validate their efficacy in different types of tumors. The ongoing clinical trials investigating the combination treatment with galunisertib are shown in Table 2. Additionally, a combined immunotherapy of ICB and TGF-β blockade is also promising. In a murine model of metastatic intestinal tumor, the combination of anti-TGF-β and anti-PD-L1 showed an impressive antitumor response compared to either monotherapy 360. Currently, new approaches for simultaneous inhibition of TGF-β and PD-L1 are under evaluation. Bintrafusp alfa (also known as M7824) is a recently developed anti-PD-L1/ TGF-βRII fusion protein, which showed superior inhibition of

tumor growth in multiple murine models 361,362. Other fusion drugs such as anti-CTLA4-TGF-βRII also showed promising efficacy in stimulating antitumor responses 363. Several early-phase trials showed encouraging clinical efficacy and manageable safety in different types of cancers 364-366. Several ongoing clinical trials are testing the efficacy and safety of bintrafusp alfa (Table 2). Wnt inhibitors including ETC-1922159 and Ipafricept (also known as OMP-54F28) were also employed in clinical control of various types of solid tumors 367-370. Ipafricept combined with chemotherapy showed reasonable tolerance in early-phase clinical trials, and remains a therapeutic target of interest 368,369,371. ETC-1922159 is now under assessment in an ongoing clinical trial to test its efficacy as a pembrolizumab combination (Table 2).With other therapyThe close interaction between inflammatory TME and EMT is well established and is regarded as a promising target for therapeutic use 15. Since TCP drives a highly inflamed TME, neutralizing monoclonal antibodies targeting IL-8 were developed to reverse TCP. HuMax-IL8 (also known as BMS-986253), a neutralizing anti-IL8 antibody that was designed to sequester IL-8 signaling, successfully inhibited the mesenchymal transition in human TNBC cell lines in vitro. Blockade of IL-8 enhanced the tumor cell-killing effect mediated by NK and T cells. Another in vivo study in NSCLC found that IL-8 neutralizing antibodies reversed the tumor plasticity and sensitized tumor cells to erlotinib. Currently, multiple ongoing clinical trials in phase I/II are examining the safety and efficacy of HuMax-IL8 as a combination therapy in various cancer types, including prostate and pancreatic cancer, HCC, NSCLC, and other solid tumors (Table 3). SX-682, another novel drug targeting the inflammatory EMT, was proposed to prevent polymorphonuclear (PMN)-MDSC

migration and cooperate well with ICB treatment 372,373. Early-phase clinical trials of SX-682 in combination with ICBs (e.g., pembrolizumab and nivolumab) are currently recruiting participants (Table 3). Hypoxia signaling contributes significantly to EMT and subsequent therapy resistance. Hypoxia-induced factor (HIF), a transcriptional regulator, was reported to be recruited to the hypoxia-responsive element of several EMT-related genes, including Twist, Snail, Vimentin, and TGF-β 374. HIF-1α promoted EMT via multiple signaling pathways including PI3K/Akt, Notch, Wnt, and NF-κB 349,375. Treatments targeting hypoxia pathway are deemed as promising blockades against TCP. For example, by blocking the nuclear translocation of HIF-1α, 6-gingerol successfully inhibits the EMT pathway and suppresses proliferation and metastasis of lung cancer 374. Salidroside enhances antitumor efficacy of platinum drugs in HCC by promoting the degradation of HIF-1α, thereby inhibiting the EMT of HCC cells 376. Similarly in lung cancer, by inhibiting hypoxia-activated NF-κB signaling pathway and EMT, a ginseng-extracted 20(R)-Ginsenoside (Rg3) increases the susceptibility of cancer cells to cisplatin treatment 349. Conclusion and perspectivesHere, we present a comprehensive description of TCP development and cancer cell evasion from targeted therapy. We delineate the non-genetic mechanisms of targeted drug-induced TCP in various types of tumors and provide insights into TCP contribution to acquired drug resistance. Owing to the rapid development of experimental techniques, dynamic changes of tumor cells in response to treatments can be observed in high resolution images. The ceaseless change of tumor cells indicates that living things even as simple as single cells show amazing adaptability under ambient pressure. Continuous monitoring of phenotypic switching can be of great help in designing specific dynamic

therapeutic regimens, and developing such monitoring techniques is worth exploring. We have been able to look into some pathways, like EMT, CSC, and transdifferentiation, that facilitate transformation of tumor cells; however, we still cannot visualize all the mechanisms contributing to TCP. Research and development for new treatment is never-ending. As an old saying goes, a coin has two sides. In spite of TCP-related therapy escape, TCP also allows therapeutic reprogramming and possibly reversal of tissue attrition. In this review, we summarize new therapeutic strategies that aim to inhibit or reverse TCP, and discuss related combination treatment regimens. We note that regulation of plasticity-related TME with ICB or direct imposition of EMT-modulating agents will not bring revolutionary improvement in cancer treatment. However, each small step forward against cancer is worth trying, and the concept of TCP remains valuable for further development of novel therapeutic strategies. Inhibition of unexpected TCP is theoretically feasible by holding the tumor cell unchanged. On the other hand, effective manipulation of TCP also holds great potential, and converting diverse tumor cells into a unified and susceptible state could be an alternative. The basket trial design should be continued to probe new treatment opportunities for different types of cancers, and special attention should be given to searching for new targets to better manipulate TCP.As for future research on TCP, modern technologies could be of great help. For example, omics technique and capacity of dealing with “big data” has largely broadened our knowledge of the molecular landscape of tumor tissues. Recently developed technologies could enable a simultaneous acquisition of multiple omics data at a single-cell level, and several analytic models have been established to interpret the high-dimensional multimodal

data377,378. Single-cell level multi-omics could precisely trace the therapy-induced responses of each cells in heterogeneous tumors from the aspects of transcriptomics, proteomics, epigenomics, even in combination with their spatial distribution. The innovative analytical modalities provide deep and comprehensive information at single-cell resolution, and could help to explore yet unrecognized cellular activity both intracellularly and intercellularly. In addition, single-cell techniques could also help to identify biomarkers to determine TCP, tumor response to targeted therapy, as well as prognosis379. Artificial intelligence could also be employed to capture the underlying regularities of such unwieldly mass of data. It was expected that one day, with the help of big data and the corresponding interpreting capacity, complete cures could be achieved by personalized treatment strategies decided by molecules at single-cell level. In the face of such capricious, unpredictable and threatening disease, scientific research will be always on the way to distill the complexity of cancer into an increasingly logical science. The attempts to get a better understanding of TCP in targeted therapy hold great promise for future blockade against therapy evasion, which may promote realization of long-lasting curative effects and even complete cure in cancer patients. Ongoing clinical trials using conventional treatments combined with plasticity-regulating treatment also indicate new opportunities for achieving the ultimate goal of complete cancer cure. Figure legends Figure 1. Changes in acquired therapy resistance induced by treatment initiation and discontinuation, with different scenarios employed to interpret the development of the therapy-resistant phenotype. According to the Darwinian selection model, drug-tolerant persistors (DTPs) that originated from the primary tumor tissue are selected and enriched by treatments. The Lamarckian

induction model regards the acquired drug-indifferent phenotype as the result of tumor cell adaptation to treatments that leads to formation of induced DTPs. The coexisting model suggests that formation of both primary and induced DTPs occur and together contribute to the acquired therapy resistance. Figure 2. Therapeutic resistance involvement in epigenetic modifications and EMT/MET. Figure 2A. Schematic representation of therapy-induced drug resistance through chemical epigenetic modifications. 6 Four core histone proteins (H2A, H2B, H3, and H4) can be diversely modified by multiple enzymes that result in methylation, acetylation, phosphorylation, or ubiquitylation. DNA can be methylated at the 5-carbon of the cytosine base to form 6 5-methylcytosine (5-mC), which can be further oxidized to form 5-hydroxymethyl cytosine (5-hmC). Enzymes involved in the epigenetic modifications of either histone or DNA can be utilized as potential future therapeutic targets against tumor cell plasticity. 45 K, Lysine; S, Serine; R, Arginine; T, Threonine; me, methylation; ac, acetylation; ub, ubiquitination; 8 P, phosphorylation; HAT, histone acetyltransferase; HMT, histone methyltransferase; HDAC, histone deacetylase; HDM, histone demethylase; TRIM, tripartite mortif; MSK1, 68 mitogen- and stress-activated protein kinase 1; 10 DNMT, DNA methyltransferase; TET, ten-eleven translocation enzymes. Figure 2B. Primary tumor cells undergo EMT and MET to achieve metastasis. Primary tumor cells undergo EMT by losing epithelial traits and acquiring mesenchymal characteristics, which enables tumor cells to migrate and invade into blood or lymphatic vessels. In circulation, tumor cells exhibit a “partial EMT” within the epithelial-mesenchymal spectrum. During colonization, migratory tumor cells with mesenchymal traits undergo MET to restore epithelial characteristics and proliferate to form a secondary tumor. Therapeutic

resistance and invasiveness are higher in mesenchymal states, whereas a hybrid state of epithelial and mesenchymal indicates the highest level of stemness and capacity of self-adaptation. The process of EMT/MET is regulated by crosstalk among genomic factors, MET-TFs, and multiple signaling pathways. EMT, epithelial-mesenchymal transition; MET, mesenchymal-epithelial transition; TF, transcription factor; miR, microRNA. Figure 3. Mechanisms driving tumor cell plasticity and drug resistance. Both tumor microenvironment factors and EMT-related signaling pathways regulate the EMT process. The activation of EMT facilitates the dedifferentiation of non-cancer stem cells (CSCs) to CSC, which contributies to tumor heterogeneity. Figure 4. Therapeutic strategies targeting tumor cell plasticity. Three main strategies can be used in combination treatment against tumor cell plasticity: 1) prevent tumor cell plasticity; 2) reverse the phenotypic switching; and 3) direct therapy to target the induced therapy-resistant tumor cells. Figure 5. Combination treatments for overcoming the tumor cell plasticity induced-therapy resistance. Tumor cells evade targeted treatments by interacting with TME and via EMT programs. Inflammation, hypoxia, and immunosuppressive TME contribute to therapy evasion, and such induced-resistance could be inhibited by a combination treatment including anti-inflammation and anti-hypoxic drugs and immunosuppressive ICB regimens. EMT-modulating agents, on the other hand, could also deal with plasticity induced-therapy resistance as a combination treatment and lead to tumor cell death. TME, tumor microenvironment; EMT, epithelial-to-mesenchymal transition; ICB, immune checkpoint blockade; Rg3, ginseng-extracted 20(R)-Ginsenoside.

6058242889765355552_11002_555FDC64649ABCCBF18DEC4...

ORIGINALITY REPORT

16%	9%	13%	1%
SIMILARITY INDEX	INTERNET SOURCES	PUBLICATIONS	STUDENT PAPERS

PRIMARY SOURCES

1	www.nature.com<br>Internet Source	2%
2	Siyuan Qin, Jingwen Jiang, Yi Lu, Edouard C. Nice, Canhua Huang, Jian Zhang, Weifeng He. "Emerging role of tumor cell plasticity in modifying therapeutic response", Signal Transduction and Targeted Therapy, 2020<br>Publication	1%
3	Soufiane Boumahdi, Frederic J. de Sauvage. "The great escape: tumour cell plasticity in resistance to targeted therapy", Nature Reviews Drug Discovery, 2019<br>Publication	1%
4	worldwidescience.org<br>Internet Source	1%
5	www.ncbi.nlm.nih.gov<br>Internet Source	1%
6	Lei Guo, Yi-Tsang Lee, Yubin Zhou, Yun Huang. "Targeting epigenetic regulatory machinery to	1%

overcome cancer therapy resistance",
Seminars in Cancer Biology, 2021
Publication

7	www.science.gov<br>Internet Source	<1 %
8	www.frontiersin.org<br>Internet Source	<1 %
9	opus4.kobv.de<br>Internet Source	<1 %
10	link.springer.com<br>Internet Source	<1 %
11	docobook.com<br>Internet Source	<1 %
12	Mark A. Rubin, Robert G. Bristow, Phillip D. Thienger, Caroline Dive, Marcin Imielinski. "Impact of Lineage Plasticity to and from a Neuroendocrine Phenotype on Progression and Response in Prostate and Lung Cancers", Molecular Cell, 2020<br>Publication	<1 %
13	tel.archives-ouvertes.fr<br>Internet Source	<1 %
14	Anja Wessely, Theresa Steeb, Carola Berking, Markus Vincent Heppt. "How Neural Crest Transcription Factors Contribute to Melanoma Heterogeneity, Cellular Plasticity,	<1 %

and Treatment Resistance", International Journal of Molecular Sciences, 2021
Publication

15	"Nuclear Signaling Pathways and Targeting Transcription in Cancer", Springer Science and Business Media LLC, 2014<br>Publication	<1 %
16	ww5.komen.org<br>Internet Source	<1 %
17	www.researchgate.net<br>Internet Source	<1 %
18	Chunying Zhang, Ying Lu, Qing Li, Jun Mao et al. "Salinomycin suppresses TGF-β1-induced epithelial-to-mesenchymal transition in MCF-7 human breast cancer cells", Chemico-Biological Interactions, 2016<br>Publication	<1 %
19	academic.oup.com<br>Internet Source	<1 %
20	Louise Walsh, William M. Gallagher, Darran P. O'Connor, Tríona Ní Chonghaile. "Diagnostic and Therapeutic Implications of Histone Epigenetic Modulators in Breast Cancer", Expert Review of Molecular Diagnostics, 2016<br>Publication	<1 %
21	Ann-Lii Cheng, Chiun Hsu, Stephen L. Chan, Su-Pin Choo, Masatoshi Kudo. "Challenges of	<1 %

combination therapy with immune checkpoint inhibitors for hepatocellular carcinoma", Journal of Hepatology, 2020
Publication

---

22 res.mdpi.com <1%
Internet Source

---

23 Fan Huang, François Santinon, Raúl Ernesto Flores González, Sonia V. del Rincón. "Melanoma Plasticity: Promoter of Metastasis and Resistance to Therapy", Frontiers in Oncology, 2021 <1%
Publication

---

24 www.dovepress.com <1%
Internet Source

---

25 Biomarkers in Disease Methods Discoveries and Applications, 2015. <1%
Publication

---

26 www.mdpi.com <1%
Internet Source

---

27 www.spandidos-publications.com <1%
Internet Source

---

28 www.cancertreatmentjournal.com <1%
Internet Source

---

29 Abhijeet P. Deshmukh, Suhas V. Vasaikar, Katarzyna Tomczak, Shubham Tripathi et al. "Identification of EMT signaling cross-talk and <1%

gene regulatory networks by single-cell RNA sequencing", Proceedings of the National Academy of Sciences, 2021
Publication

30 Vivian Weiwen Xue, Jeff Yat-Fai Chung, Cristina Alexandra García Córdoba, Alvin Ho-Kwan Cheung et al. "Transforming Growth Factor-β: A Multifunctional Regulator of Cancer Immunity", Cancers, 2020 <1%
Publication

31 effiloop.com <1%
Internet Source

32 Hossein Borghaei, Corey J. Langer, Luis Paz-Ares, Delvys Rodríguez-Abreu et al. "Pembrolizumab plus chemotherapy versus chemotherapy alone in patients with advanced non-small cell lung cancer without tumor PD-L1 expression: A pooled analysis of 3 randomized controlled trials", Cancer, 2020 <1%
Publication

33 Leah J. Greenspan, Brant M. Weinstein. "To be or not to be: endothelial cell plasticity in development, repair, and disease", Angiogenesis, 2021 <1%
Publication

34 Katsuno, Yoko, Samy Lamouille, and Rik Derynck. "TGF-β signaling and epithelial– <1%

mesenchymal transition in cancer progression :", Current Opinion in Oncology, 2013.
Publication

35 Anastasios Gkountakos, Giovanni Centonze, Emanuele Vita, Lorenzo Belluomini et al. "Identification of Targetable Liabilities in the Dynamic Metabolic Profile of EGFR-Mutant Lung Adenocarcinoma: Thinking beyond Genomics for Overcoming EGFR TKI Resistance", Biomedicines, 2022 <1%
Publication

36 Kazuhiko Igarashi, Hironari Nishizawa, Yuriko Saiki, Mitsuyo Matsumoto. "The transcription factor BACH1 at the crossroads of cancer biology: From epithelial–mesenchymal transition to ferroptosis", Journal of Biological Chemistry, 2021 <1%
Publication

37 scholars.uthscsa.edu <1%
Internet Source

38 ashpublications.org <1%
Internet Source

39 studyres.com <1%
Internet Source

40 www.asbmb.org <1%
Internet Source

www2.mdpi.com

41	Internet Source	<1%
42	Golnoush Dehbashi Behbahani, Nastaran Mohammadi Ghahhari, Mohammad Amin Javidi, Asghar Farzi Molan, Neda Feizi, Sadegh Babashah. "MicroRNA-Mediated Post-Transcriptional Regulation of Epithelial to Mesenchymal Transition in Cancer", Pathology & Oncology Research, 2016<br>Publication	<1%
43	Yuanyuan Zhang, Hongyan Chen, Hongnan Mo, Xueda Hu et al. "Single-cell analyses reveal key immune cell subsets associated with response to PD-L1 blockade in triple-negative breast cancer", Cancer Cell, 2021<br>Publication	<1%
44	edoc.ub.uni-muenchen.de<br>Internet Source	<1%
45	mdpi-res.com<br>Internet Source	<1%
46	Submitted to Columbia University<br>Student Paper	<1%
47	Meghan W. Dukes, Elizabeth A. Bajema, Tyler J. Whittemore, Robert A. Holmgren, Thomas J. Meade. "Delivery of Targeted Co(III)–DNA Inhibitors of Gli Proteins to Disrupt Hedgehog Signaling", Bioconjugate Chemistry, 2022	<1%

Publication

48	Saghar Yousefnia, Farzad Seyed Forootan, Shiva Seyed Forootan, Mohammad Hossein Nasr Esfahani, Ali Osmay Gure, Kamran Ghaedi. "Mechanistic Pathways of Malignancy in Breast Cancer Stem Cells", Frontiers in Oncology, 2020<br>Publication	<1%
49	ijgc.bmj.com<br>Internet Source	<1%
50	internal-journal.frontiersin.org<br>Internet Source	<1%
51	orca.cf.ac.uk<br>Internet Source	<1%
52	"Full Issue PDF", JACC: CardioOncology, 2022<br>Publication	<1%
53	"TGF-β-induced epithelial to mesenchymal transition", Cell Research, 02/2009<br>Publication	<1%
54	Lei Du, Qi Cheng, Hao Zheng, Jinming Liu, Lei Liu, Quan Chen. "Targeting stemness of cancer stem cells to fight colorectal cancers", Seminars in Cancer Biology, 2022<br>Publication	<1%
55	Yuchen Jin, Xian Qiu, Ziyan He, JunYao Wang, Ri Sa, Libo Chen. "ERBB2 as a prognostic	<1%

biomarker correlates with immune infiltrates in papillary thyroid cancer", Frontiers in Genetics, 2022
Publication

56	bjp.sagepub.unboundmedicine.com<br>Internet Source	<1 %
57	pharmaceuticalintelligence.com<br>Internet Source	<1 %
58	ruj.uj.edu.pl<br>Internet Source	<1 %
59	tessera.spandidos-publications.com<br>Internet Source	<1 %
60	www.fedoa.unina.it<br>Internet Source	<1 %
61	"Abstract", Breast Cancer Research and Treatment, 2006<br>Publication	<1 %
62	Jun Li, Hailin Yu, Meili Xi, Duan Ma, Xin Lu. "The SNAI1 3'UTR functions as a sponge for multiple migration-/invasion-related microRNAs", Tumor Biology, 2014<br>Publication	<1 %
63	Kelly J. Gordon, Mei Dong, Elizabeth M. Chislock, Timothy A. Fields, Gerard C. Blobe. "Loss of type III transforming growth factor β receptor expression increases motility and	<1 %

invasiveness associated with epithelial to mesenchymal transition during pancreatic cancer progression", Carcinogenesis, 2008

Publication

---

64 Yoko Katsuno, Dominique Stephan Meyer, Ziyang Zhang, Kevan M. Shokat, Rosemary J. Akhurst, Kohei Miyazono, Rik Derynck. "Chronic TGF-β exposure drives stabilized EMT, tumor stemness, and cancer drug resistance with vulnerability to bitopic mTOR inhibition", Science Signaling, 2019 <1%

Publication

---

65 "MRS-AACR Joint Conference on Metastasis 2010", Clinical & Experimental Metastasis, 2010 <1%

Publication

---

66 D. S. Salomon. "Review of: Metalloproteinase axes increase β-catenin signaling in primary mouse mammary epithelial cells lacking TIMP3", Breast Cancer Online, 2007 <1%

Publication

---

67 Kar, Swayamsiddha, Moonmoon Deb, Dipta Sengupta, Arunima Shilpi, Sujit Kumar Bhutia, and Samir Kumar Patra. "Intricacies of hedgehog signaling pathways: A perspective in tumorigenesis", Experimental Cell Research, 2012. <1%

Publication

---

68 Luca Carlino, Giulio Rastelli. "Dual Kinase-Bromodomain Inhibitors in Anticancer Drug Discovery: A Structural and Pharmacological Perspective", Journal of Medicinal Chemistry, 2016 <1%
Publication

69 Nikki K. Lytle, Alison G. Barber, Tannishtha Reya. "Stem cell fate in cancer growth, progression and therapy resistance", Nature Reviews Cancer, 2018 <1%
Publication

70 Ning Pu, Guochao Zhao, Hanlin Yin, Jian-ang Li et al. "CD25 and TGF-β blockade based on predictive integrated immune ratio inhibits tumor growth in pancreatic cancer", Journal of Translational Medicine, 2018 <1%
Publication

71 Wiwin Is Effendi, Tatsuya Nagano. "The Hedgehog Signaling Pathway in Idiopathic Pulmonary Fibrosis: Resurrection Time", International Journal of Molecular Sciences, 2021 <1%
Publication

72 Xie, K.. "Interleukin-8 and human cancer biology", Cytokine and Growth Factor Reviews, 200112 <1%
Publication

73 Yoshimi Arima. "Decreased expression of neurofibromin contributes to epithelialâmesenchymal transition in neurofibromatosis type 1", Experimental Dermatology, 11/2009 <1%

Publication

Exclude quotes	Off	Exclude matches	Off
Exclude bibliography	Off		
