# Supplementary material for: Tumor cell plasticity in targeted therapy-induced resistance: mechanisms and new strategies
Source: Signal Transduct Target Ther. 2023 Mar 11;8:113. doi: 10.1038/s41392-023-01383-x (PMC10008648; doi:10.1038/s41392-023-01383-x)
Supplement: Supplementary file 2 — Editing Certificate1 [file 41392_2023_1383_MOESM2_ESM.pdf]

editage

Editing Certificate

This document certifies that the manuscript listed below has been edited to ensure language and grammar accuracy and is error free in these aspects. The logical presentation of ideas and the structure of the paper were also checked during the editing process. The edit was performed by professional editors at Editage, a division of Cactus Communications. The author's core research ideas were not altered in any way during the editing process. The quality of the edit has been guaranteed, with the assumption that our suggested changes have been accepted and the text has not been further altered without the knowledge of our editors.

MANUSCRIPT TITLE

Tumor cell plasticity in targeted therapy-induced resistance: mechanisms and new strategies

AUTHORS

Zhen-Duo Shi,Kun Pang, Zhuo-Xun Wu, Yang Dong, Lin Hao, Jia-xin Qin, Wei Wang, Zhe-Sheng Chen, Cong-Hui Han

ISSUED ON

October 21, 2022

JOB CODE

DESAO_5

THE EDITAGE Promise QUALITY GUARANTEE

Vikas Narang

Vikas Narang
Chief Operating Officer - Editage

editage

Editage, a brand of Cactus Communications, offers professional English language editing and publication support services to authors engaged in over 500 areas of research. Through its community of experienced editors, which includes doctors, engineers, published scientists, and researchers with peer review experience, Editage has successfully helped authors get published in internationally reputed journals. Authors who work with Editage are guaranteed excellent language quality and timely delivery.

GLOBAL :
+1(833) 979-0061 | request@editage.com
