# Supplementary material for: Tumor cell plasticity in targeted therapy-induced resistance: mechanisms and new strategies
Source: Signal Transduct Target Ther. 2023 Mar 11;8:113. doi: 10.1038/s41392-023-01383-x (PMC10008648; doi:10.1038/s41392-023-01383-x)
Supplement: Supplementary file 3 — Editing Certificate2 [file 41392_2023_1383_MOESM3_ESM.pdf]

UNIWINSCI

730 P.O. Box, Piscataway, NJ 08854. Tel. 302-990-2687. Email: uniwinsci@yahoo.com

CERTIFICATE

Tumor cell plasticity in targeted therapy-induced resistance: mechanisms and new strategies

Service date: September 9, 2022

This is to certify that the above manuscript has been copyedited by a native English speaker in the UNIWINSCI Team in the United States. The UNIWINSCI INC. has experts in all fields of science and has been serving scientists around the world for their writing and editing needs since 2008.

Dona Cooper, MD, PhD

President

The UNIWINSCI Team
